# Cost-effective solutions for high-throughput enzymatic DNA methylation sequencing

**DOI:** 10.1371/journal.pgen.1011667

**Published:** 2025-05-22

**Authors:** Amy Longtin, Marina M. Watowich, Baptiste Sadoughi, Rachel M. Petersen, Sarah F. Brosnan, Kenneth Buetow, Qiuyin Cai, Cayo Biobank Research Unit, Michael D. Gurven, James P. Higham, Heather M. Highland, Yi-Ting Huang, Hillard Kaplan, Thomas S. Kraft, Yvonne A. L. Lim, Jirong Long, Amanda D. Melin, Michael J. Montague, Jamie Roberson, Kee Seong Ng, Michael L. Platt, India A. Schneider-Crease, Jonathan Stieglitz, Benjamin C. Trumble, Vivek V. Venkataraman, Ian J. Wallace, Jie Wu, Noah Snyder-Mackler, Angela Jones, Alexander G. Bick, Amanda J. Lea

**Affiliations:** 1 Department of Biological Sciences, Vanderbilt University, Nashville, Tennessee, United States of America; 2 Evolutionary Studies Initiative, Vanderbilt University, Nashville, Tennessee, United States of America; 3 School of Life Sciences, Arizona State University, Tempe, Arizona, United States of America; 4 Center for Evolution and Medicine, Arizona State University, Tempe, Arizona, United States of America; 5 Departments of Psychology & Philosophy, Neuroscience Institute, Center for Behavioral Neuroscience, and the Language Research Center, Georgia State University, Atlanta, GeorgiaUnited States of America; 6 Division of Epidemiology, Department of Medicine, Vanderbilt University Medical Center, Nashville, Tennessee, United States of America; 7 Department of Anthropology, University of California, Santa Barbara, California, United States of America; 8 Department of Anthropology, New York University, New York, New York, United States of America; 9 New York Consortium in Evolutionary Primatology, New York, New York, United States of America; 10 Department of Epidemiology, University of North Carolina at Chapel Hill, Chapel Hill, North Carolina, United States of America; 11 Vanderbilt Genetics Institute, Vanderbilt University School of Medicine, Nashville, Tennessee, United States of America; 12 Institute for Economics and Society, Chapman University, Orange, California, United States of America; 13 Department of Anthropology, University of Utah, Salt Lake City, Utah, United States of America; 14 Department of Parasitology, Faculty of Medicine, Universiti Malaya, Kuala Lumpur, Malaysia; 15 Centre for Malaysian Indigenous Studies (CMIS), Universiti Malaya, Kuala Lumpur, Malaysia; 16 Department of Anthropology & Archaeology, University of Calgary, Calgary, Alberta, Canada; 17 Department of Medical Genetics, University of Calgary, Calgary, Alberta, Canada; 18 Alberta Children’s Hospital Research Institute, Calgary, Alberta, Canada; 19 Department of Neuroscience, Perelman School of Medicine, University of Pennsylvania, Philadelphia, Pennsylvania, United States of America; 20 Department of Medicine, Faculty of Medicine, Universiti Malaya, Kuala Lumpur, Malaysia; 21 Department of Psychology, School of Arts and Sciences, University of Pennsylvania, Philadelphia, Pennsylvania, United States of America; 22 Marketing Department, Wharton School of Business, University of Pennsylvania, Philadelphia, Pennsylvania, United States of America; 23 School of Human Evolution and Social Change, Arizona State University, Tempe, Arizona, United States of America; 24 Department of Social and Behavioral Sciences, Toulouse School of Economics, Institute for Advanced Study in Toulouse, Université Toulouse Capitole, Toulouse, France; 25 Institute of Human Origins, Arizona State University, Tempe, Arizona, United States of America; 26 Department of Anthropology, University of New Mexico, Albuquerque, New Mexico, United States of America; 27 Division of Genetic Medicine, Department of Medicine, Vanderbilt University Medical Center, Nashville, Tennessee, United States of America; The University of Edinburgh MRC Human Genetics Unit, UNITED KINGDOM OF GREAT BRITAIN AND NORTHERN IRELAND

## Abstract

Characterizing DNA methylation patterns is important for addressing key questions in evolutionary biology, development, geroscience, and medical genomics. While costs are decreasing, whole-genome DNA methylation profiling remains prohibitively expensive for most population-scale studies, creating a need for cost-effective, reduced representation approaches (i.e., assays that rely on microarrays, enzyme digests, or sequence capture to target a subset of the genome). Most common whole genome and reduced representation techniques rely on bisulfite conversion, which can damage DNA resulting in DNA loss and sequencing biases. Enzymatic methyl sequencing (EM-seq) was recently proposed to overcome these issues, but thorough benchmarking of EM-seq combined with cost-effective, reduced representation strategies is currently lacking. To address this gap, we optimized the Targeted Methylation Sequencing protocol (TMS)—which profiles ~4 million CpG sites—for miniaturization, flexibility, and multispecies use. First, we tested modifications to increase throughput and reduce cost, including increasing multiplexing, decreasing DNA input, and using enzymatic rather than mechanical fragmentation to prepare DNA. Second, we compared our optimized TMS protocol to commonly used techniques, specifically the Infinium MethylationEPIC BeadChip (n = 55 paired samples) and whole genome bisulfite sequencing (n = 6 paired samples). In both cases, we found strong agreement between technologies (R^2^ = 0.97 and 0.99, respectively). Third, we tested the optimized TMS protocol in three non-human primate species (rhesus macaques, geladas, and capuchins). We captured a high percentage (mean = 77.1%) of targeted CpG sites and produced methylation level estimates that agreed with those generated from reduced representation bisulfite sequencing (R^2^ = 0.98). Finally, we confirmed that estimates of 1) epigenetic age and 2) tissue-specific DNA methylation patterns are strongly recapitulated using data generated from TMS versus other technologies. Altogether, our optimized TMS protocol will enable cost-effective, population-scale studies of genome-wide DNA methylation levels across human and non-human primate species.

## Author summary

DNA methylation profiling is important for understanding key questions in biology, but current techniques can be expensive and have technical limitations. Enzymatic methyl sequencing (EM-seq) was proposed as a potential solution, but thorough testing is still needed. In this study, we optimized a new method (Targeted Methylation Sequencing or TMS) to make it more cost-effective and flexible, and applied it to multiple species. We tested modifications to increase sample multiplexing, reduce DNA input, and use enzymatic fragmentation. We compared our optimized TMS protocol to common methylation profiling techniques and found strong agreement in DNA methylation levels. We also successfully applied the optimized TMS protocol to three non-human primate species. Finally, we show that common analyses of DNA methylation data produce similar results using TMS data versus data from other technologies. Together, we hope this work will enable cost-effective, population-scale DNA methylation profiling across human and non-human species.

## Introduction

Understanding variation in DNA methylation levels across tissues, the lifespan, disease states, and populations is important for addressing key questions in biology. DNA methylation—the covalent addition of methyl groups to cytosines—is a semi-malleable and environmentally-responsive epigenetic modification involved in gene regulation in many species, including our own [1]. Because DNA methylation moderates gene expression throughout the life course, it is critical for processes such as development [2–4], cell programming [5], tissue specificity [6], aging [7–11], and disease progression [12–14]. For example, changes in DNA methylation are considered a “hallmark” of the aging process, with most studies reporting age-associated gains in methylation in hypomethylated regions (e.g., promoters and transcribed regions) and age-associated losses in methylation in hypermethylated regions (e.g., heterochromatic regions, Polycomb-repressed regions) [15–17]. These age-related patterns are so consistent that DNA methylation variation has been used to construct molecular clocks that reliably predict chronological age [18,19]. Further, because DNA methylation is known to respond to environmental inputs, it has been implicated as a mechanism through which diverse environmental exposures can impact long-term physiology and health (e.g., famine [20–24], psychosocial stress [25–29], or infection [30–33]).

To profile genome-wide DNA methylation at scale, most studies rely on reduced representation methods: human studies have largely favored microarrays, while non-human studies have favored reduced representation bisulfite sequencing (RRBS) due to the historical lack of species-specific microarrays (though recent work has led to the development of the Infinium Mouse DNA Methylation BeadChip as well as the Mammalian Methylation Array) [34–36]. Both RRBS and microarrays quantify DNA methylation at a subset (1–5%) of CpGs in the genome, and thus provide a cost-effective strategy relative to genome-wide assays (e.g., whole genome bisulfite sequencing (WGBS)). For example, the Infinium MethylationEPIC v2.0 BeadChip, or EPIC array, covers ~ 930K CpG sites including functional elements identified by the ENCODE project [37], DNase hypersensitive sites, and putatively important sites for human disease and development [38,39]. In contrast, RRBS fragments DNA using the Msp1 enzyme that cuts DNA at CCGG motifs, which following size selection, enriches for 1–5% of the genome with high CpG content such as CpG islands and gene bodies [34,40]. Importantly, both microarrays and RRBS rely on sodium bisulfite, which converts unmethylated cytosines to thymine while leaving methylated cytosines protected from conversion. This chemical reaction requires high pHs and temperatures, which can cause unwanted DNA fragmentation and damage, especially to unmethylated cytosines [41]. Ultimately, such damage can create difficulties during library preparation as well as biases in the downstream data [41–43].

Enzymatic methyl sequencing (EM-seq) offers a useful alternative to bisulfite sequencing with several key benefits: EM-seq relies on enzymatic rather than chemical conversion of unmethylated cytosines to thymine, resulting in substantially less DNA damage [42]. As a result, whole genome EM-seq has been shown to recover more CpGs sites, have lower duplication rates, have better between-replicate correlations, and require less DNA input than WGBS [42]. However, existing EM-seq benchmarked protocols rely on whole genome rather than reduced representation strategies, hindering their adoption especially for population-scale studies. To address this gap, Twist Biosciences recently created a hybrid capture panel that targets ~4 million CpG sites in the human genome and is compatible with EM-seq. The Twist methylation capture reaction uses ~ 550k probes to target functionally relevant CpG sites (e.g., those in enhancers, gene bodies, and near transcription start sites) and to cover ~95% of CpG sites included on the widely used EPIC array [44–47]. Off the shelf, this protocol is similar or lower in cost to existing reduced representation approaches. However, we note that total cost for any sequencing-based approach will depend on the desired coverage (i.e., the average number of reads that cover each CpG site); best practices for average per CpG coverage are still debated, but most studies recommend at least 20x [48–51]. Increased coverage will increase the precision of DNAm estimates, and thus to some degree the desired coverage depends on the anticipated effect size.

**Fig 1:**
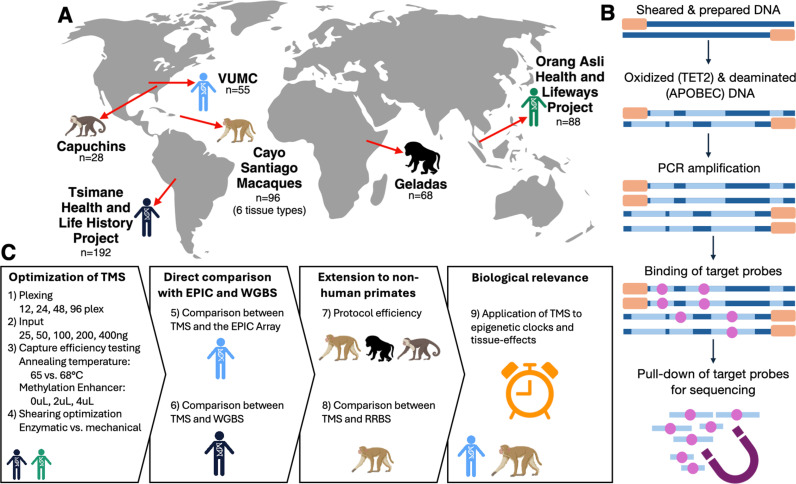
Experimental design and study populations. **[A]** To optimize the TMS protocol, we used samples from three human and three NHP populations: the Tsimane of Bolivia, a Vanderbilt University Medical Center cohort, the Orang Asli of Malaysia, rhesus macaques from Cayo Santiago in Puerto Rico, tufted capuchins from captive sites throughout the United States, and gelada monkeys from Ethiopia. Created using BioRender. **[B]** The TMS protocol begins with DNA fragmentation and adapter ligation. Next, two enzymes, TET2 and APOBEC, are used to oxidize and deaminate the DNA. TET2 recognizes methyl groups attached to cytosines and converts them to Ca/g. APOBEC follows TET2 and converts the unmethylated cytosines to uracils. Following PCR amplification (which converts uracils to thymines), hybrid capture is used to enrich for targeted regions of the genome. Samples are then assayed via high throughput sequencing. Created using Microsoft Powerpoint. **[C]** Overview of experiments and analyses. The samples used for each set of experiments are noted by a population-specific icon. Icons from Biorender, OpenClipArt, and Microsoft Powerpoint.

Here, we aimed to develop and benchmark an optimized and further cost-reduced version of the targeted methylation sequencing (TMS) approach suitable for population-scale studies, including both human and non-human primate (NHP) studies (Fig 1A). To do so, we built upon the off the shelf TMS protocol (Fig 1B), which recommends 8 plexing of samples per capture reaction and 200 ng of DNA input, and tested four multiplexing strategies (12, 24, 48, and 96 plex, using 200 ng of sample input; Fig 1C). We also tested five DNA input amounts (25, 50, 100, 200, and 400ng, using the 12-plex strategy) and other minor protocol modifications such as varying the annealing temperature during hybrid capture and varying the method used for DNA fragmentation (Fig 1C). Following optimization, we assessed: 1) the robustness of our protocol through a direct comparison with the EPIC array and WGBS; 2) the extension of optimized TMS for use in NHP species; and 3) the ability of our protocol to recapitulate biological results (epigenetic age estimates and identification of tissue-specific patterns) obtained from data generated using other technologies (see Table 1 for sample sizes and sample information; Fig 1C). Overall, we found that we were able to miniaturize and optimize the TMS protocol to ~USD 80 per sample, while maintaining data quality and comparability to existing methods. In total, our protocol provides coverage of approximately four times as many CpG sites relative to the EPIC array at one fourth the cost—a ~ 16-fold gain in the data-to-price ratio (S1 Table).

**Table 1:** Study populations and sample information for each experiment (names of experiments are as described in Results). F = female, M = male, VUMC = Vanderbilt University Medical Center. See also S2 Table for sample metadata and read depth.

Population (species)	Tissue type	Sample size	Sex	Mean sample age (range)	Experiment
Tsimane (human)	Whole blood	n = 192	103 F; 89 M	49.6 years old (18.0–83.6)	1, 2, 3, 6, 8
VUMC (human)	Whole blood	n = 55	31 F; 24 M	Not available	5, 9
Orang Asli (human)	White blood cells	n = 88	46 F; 42 M	35.3 years old (18–78)	4
Rhesus macaque (*Macaca mulatta*)	Heart (16), kidney (16), adrenal (16), spleen (16), lung (16), liver (16)	n = 96	39 F; 57 M	10.57 years old (3.18–19.93)	7, 8, 9
Gelada (*Theropithecus gelada*)	Whole blood	n = 68	21 F; 47 M	Unknown; all animals >5 years old	7
Capuchin (*Sapajus apella*)	Whole blood	n = 28	19 F; 9 M	19.4 years old (9.0–41.0)	7
**Total**		**n = 527**			

## Results

### Data quality is robust to a range of multiplexing strategies, input amounts, and protocol modifications

#### Experiments 1 & 2: Varying multiplexing strategies and input amounts.

**Fig 2:**
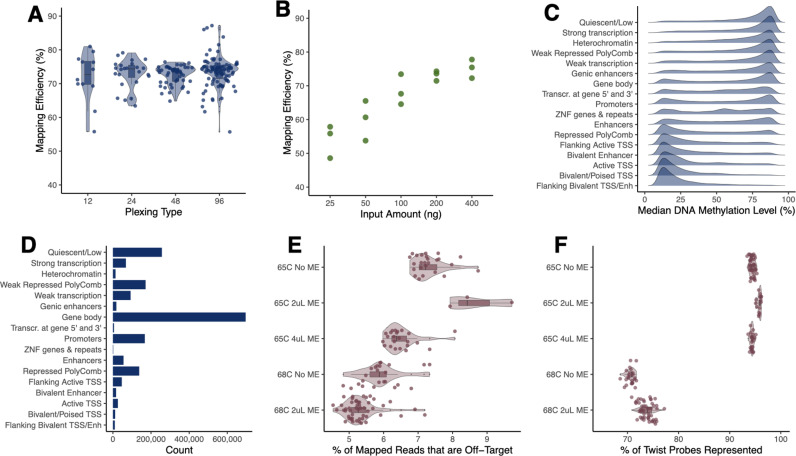
Optimized TMS produces high-quality DNA methylation data across a range of plexing strategies, input amounts, and protocol modifications. **[A]** High (>70%) mean mapping efficiency across plexing strategies. Each point represents a sample within a plexing strategy and the y-axis represents the percent of reads uniquely mapped per sample. **[B]** Mapping efficiency increases as input amount increases. Each point represents a 12-plex pool made with varying DNA input amounts per sample, the y-axis represents the percent of reads uniquely mapped per sample. **[C]** Distribution of median DNA methylation levels for CpG sites located within different chromHMM genomic annotations; annotations from NIH Roadmap Epigenomics and data from the 96-plex, 200 ng input from experiment 1. **[D]** The total number of CpG sites falling within different chromHMM genomic annotations (using data from the 96-plex, 200 ng input from experiment 1). **[E]** Percent of reads that are not within the Twist probe set (i.e., off-target reads) following protocol modifications to annealing temperature and methylation enhancer (ME) volume. For each set of protocol conditions, the x-axis represents the percent of mapped reads that do not overlap with the Twist probe set. **[F]** Percent of Twist probes that are represented within each dataset following protocol modifications to adjust the annealing temperature and ME volume. For each set of protocol conditions, the x-axis represents the percentage of Twist probes that were represented by at least 1 read.

Using DNA from a human population in Bolivia (Tsimane, see [52]), we tested four multiplexing strategies (12, 24, 48, and 96 plex, using 200ng of DNA sample input) and five DNA input amounts (25, 50, 100, 200, and 400ng, using the 12-plex strategy). Raw quality control metrics such as percent CHH methylation (a proxy for the rate at which unmethylated cytosines are converted to thymine) and mapping efficiency (percent of reads uniquely mapped to the genome) were high for all samples. Mapping efficiency was consistent across plexing strategies (average mapping efficiency: 12-plex = 71.9%, 24-plex = 72.9%, 48-plex = 72.5%, and 96-plex = 73.5%; ANOVA: F-value = 0.843, p-value = 0.472; Fig 2A) but affected by input amount, with higher DNA input having greater mapping efficiency (ANOVA: F-value = 13.57, p-value < 0.001, Fig 2B and S3 Table). CHH methylation was consistently well below 1%, indicative of high conversion rate across all plexing and input strategies (range = 0.1-0.27%; S1 Fig, and S4 and S5 Tables) [53].

After filtering for CpG sites with>5x coverage that were within the Twist probe set (+/- 200 bp) and that were covered in the majority of samples in a given experiment, we retained an average of 4,197,008 CpG sites (s.d. = 546,767) across plexing experiments and 4,051,941 CpG sites (s.d. = 93,106) across input experiments (S6 and S7 Tables). On average, this represented 96.42% and 92.19% coverage of the TMS probe set across the plexing and input experiments, respectively (S8 and S9 Tables). Across experiments, we found average coverage of targeted CpG sites to be far greater than our minimum required coverage of 5x, ranging from 21-89x across datasets (S2 Fig and S2 Table). In addition to consistently recovering the expected set of CpGs, we also observed repeatable methylation levels across the plexing and input experiments (all R^2^ > 0.99; S10 and S11 Tables). The CpGs covered by our experiments were distributed across diverse genomic annotations, and the median DNA methylation levels within a given annotation displayed expected patterns (Fig 2C and 2D) [54]. For example, we observed high levels of methylation in quiescent and heterochromatin regions and low levels of methylation in promoters and transcribed regions.

#### Experiments 3 & 4: Optimizing capture efficiency and DNA fragmentation strategies.

In experiments 1 and 2, we used the recommended 65°C annealing temperature during the hybrid capture step—where prepared DNA is bound to the capture probe set to select CpG sites of interest—and the recommended 2uL of methylation enhancer, which increases the efficiency of this reaction. Here, we found that ~3/4 of all of our mapped reads were “on-target”, meaning that they overlapped with the designed probe set and represented successful hybrid capture (S8 and S9 Tables). This suggests that ~ ¼ of reads are “off target” and randomly distributed across the genome rather than within our regions of interest. We therefore performed a third experiment using Tsimane DNA to test two protocol modifications that might decrease the off-target proportion: we increased the annealing temperature (testing 65°C or 68°C) and we varied the amount of methylation enhancer (testing 0uL, 2uL, or 4uL). We note that similar previous work has reported on-target read percentages of 75–85% [55–57], suggesting the capture reaction will likely never be completely efficient.

In experiment 3, we found that increasing the annealing temperature from 65°C to 68°C resulted in a lower proportion of off-target reads (ANOVA: F-value = 84.2, p-value < 0.0001; Figs 2E and S4, and S12 Table). Across samples annealed at 65°C, an average of 78.5% of reads were on-target, while this number rose to 84.2% at 68°C. However, this increase in capture efficiency came at a cost to the breadth of CpG sites covered: across samples annealed at 65°C, we observed coverage of on average 92.0% of the probe set, while this number fell to 72.2% for samples annealed at 68°C (Fig 2F, and S13 and S14 Tables). This suggests that higher annealing temperatures lead to greater but more specific binding during the hybrid capture step, and thus the increased capture efficiency comes at the expense of recovering all the expected CpG sites. In general, we did not find meaningful differences across methylation enhancer amounts and we therefore excluded this reagent from downstream experiments (Fig 2E and 2F). Given the loss of certain genomic regions at 68°C, downstream experiments focused on a 65°C annealing temperature.

We next performed a fourth experiment focused on protocol optimization, in which we varied the strategies used to fragment genomic DNA prior to EM-seq library preparation: specifically, we tested mechanical fragmentation via Covaris sonication against enzymatic fragmentation with the NEBNext UltraShear reagent. Mechanical fragmentation is the current standard approach but is expensive, requires special equipment, and is labor intensive. Conversely, enzymatic fragmentation is cheaper, does not require special equipment, and is more compatible with automation. For experiments 3 and 4, we used the 96-plex strategy and 200 ng of sample input, since experiments 1 and 2 suggested that data quality does not suffer from higher plexing strategies. These experiments used DNA from a human population in Malaysia, the Orang Asli [58].

Enzymatic fragmentation resulted in a similar number of covered sites as was previously observed with mechanical fragmentation (n = 4,591,123 and 4,523,981 filtered CpG sites for the 10 and 20 minute protocols, respectively). Average site-specific methylation levels were also highly concordant between approaches (mechanical versus 10 min enzymatic: R^2^ = 0.9875; mechanical versus 20 min enzymatic: R^2^ = 0.9876; 10 min versus 20 min enzymatic: R^2^ = 0.9944; S5 Fig). This was also true when we focused on a subset of DNA samples processed using both methods (n = 3; mechanical versus 10 min enzymatic: average R^2^ = 0.971; mechanical versus 20 min enzymatic: average R^2^ = 0.971; 10 min versus 20 min enzymatic: average R^2^ = 0.987, S15 Table). From these experiments, we concluded that enzymatic fragmentation can be substituted into the protocol with no loss to data quality.

We also used these data, which represent our “best” protocol (96-plex, 200ng input, 65°C annealing, no methylation enhancer, enzymatic fragmentation), to understand two critical aspects of experimental design—how many reads one would need to generate to achieve a given mean (or median) coverage per CpG site (S6 Fig) and how this mean coverage impacts power to detect differential methylation (S3 Fig). In general, we observe a ~ 1:1 relationship between the number of mapped, paired end reads (in millions) and mean coverage, such that 20 million mapped paired end (40 million total reads) translates to ~20x mean coverage (or ~14x median coverage) per CpG site. Using simulations [49,59] of datasets of different sizes (n = 100, 200, 400) and mean coverages (20x, 40x, and 80x), we found that increasing coverage can provide power gains for smaller sample sizes, but in larger datasets increasing coverage will matter less as power is derived from the overall sample size rather than gains in precision (S3 Fig).

### Epigenomic profiles measured with TMS recapitulate those measured with the EPIC array and WGBS

#### Experiment 5: Comparison of TMS to the EPIC array.

**Fig 3:**
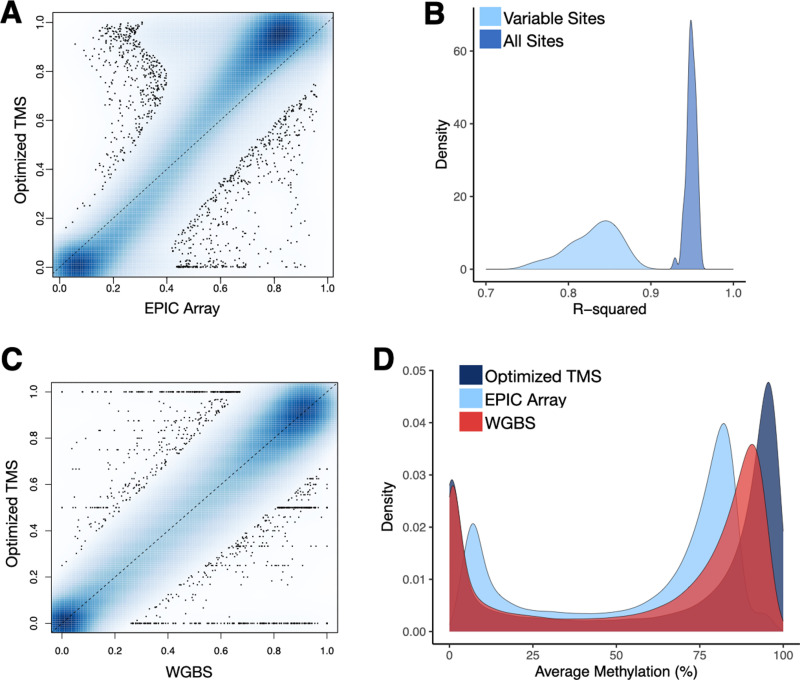
Optimized TMS recapitulates DNA methylation levels measured with the EPIC array and WGBS. **[A]** Correlation in DNA methylation levels for EPIC array versus TMS (R^2^ = 0.97). Each point represents the DNA methylation level of a given CpG averaged across 6 samples measured using the EPIC array (x-axis) and 96-plex, 200 ng input TMS (y-axis). The R^2^ value was generated using linear modeling. Sites were filtered to>5X coverage in >75% of samples within each technology. **[B]** Histogram of R^2^ values calculated for each individual sample (i.e., comparing per CpG DNA methylation levels measured on both technologies for a given sample). R^2^ values are provided when all CpG sites common to both technologies are included, as well as when only variably methylated CpG sites are included. **[C]** Correlation in DNA methylation levels for WGBS versus TMS (R^2^ = 0.9871). Each point represents the DNA methylation level of a given CpG averaged across 6 samples measured using WGBS (x-axis) and 96-plex, 200 ng input TMS (y-axis). The R^2^ value was generated using linear modeling. Sites were filtered to>5X coverage in >75% of samples within each technology. **[D]** Density plot of the average DNA methylation levels detected for common sites between the three technologies (713,282 sites). Notably, the EPIC array is biased against DNA methylation levels of 100%, as previously observed [51] and explained by the equation used to calculate beta values.

To ensure that TMS could perform comparably to the most popular current reduced-representation technology (the EPIC array), we generated paired data for 55 samples using both platforms (and following the 96-plexing, 200 ng input TMS protocol from experiment 1). After filtering, we analyzed 682,295 CpG sites common to both technologies, and found high concordance between per-site DNA methylation levels averaged across all individuals in the dataset (R^2^ = 0.97; Fig 3A). We also examined correlations between the two technologies when we subsetted to 1) variably methylated CpG sites (i.e., sites with methylation levels >10% and <90%; mean R^2^ = 0.83; Fig 3B); 2) CpG islands, shores, and shelves (R^2^ = 0.97, 0.97, 0.94, respectively); 3) hypomethylated (<50% average methylation; R^2^ = 0.89) regions; 4) hypermethylated (>50% average methylation; R^2^ = 0.70) regions; and 5) particular EPIC v2 array probe types (S7 and S8 Figs). Because methylation patterns are relatively consistent across individuals for many regions of the human genome, we also confirmed that these correlations were higher for EPIC-TMS data generated from the same sample compared to EPIC-TMS comparisons made between random pairs of samples (mean R^2^ for all sites: 0.95 versus 0.93 for random sample pairs, mean R^2^ for variable sites only: 0.83 versus 0.75 for random sample pairs; S9 Fig).

Of note, the analyses described above reconfirmed a known bias in the EPIC array data [51,60], which does not allow for methylation levels of 100%. This is because EPIC-derived DNA methylation levels are represented as beta values, calculated as the ratio of the intensity of the methylated bead type to the total locus intensity plus an offset value. Due to the addition of the offset value, beta values of 1 are mathematically impossible. As a result, the correlation between average TMS- and EPIC-measured DNA methylation levels is slightly off the x = y line (Fig 3A) and correlations are much lower than the genome-wide average for hypo- as well as hyper-methylated regions.

#### Experiment 6: Comparison of TMS to WGBS.

For further validation, we also generated WGBS data for 6 Tsimane samples included in experiment 3 (96-plexing, 200 ng input, 65°C annealing temperature, no ME, mechanical fragmentation). After filtering and merging with the TMS data, we retained 3,078,771 CpG sites covered by both the TMS and WGBS approaches. For these sites, the average methylation levels observed across technologies was highly correlated (R^2^: 0.9871; Fig 3C). We also found that the genome-wide distribution of DNA methylation levels derived from WGBS was more similar to TMS than to the EPIC array, specifically in that it included many sites with average methylation levels of 100% or close to 100%, as expected (Figs 3D and S10).

### TMS can be effectively applied to non-human primate species

#### Experiment 7: Applying TMS to tufted capuchin, rhesus macaque, and gelada samples.

**Fig 4:**
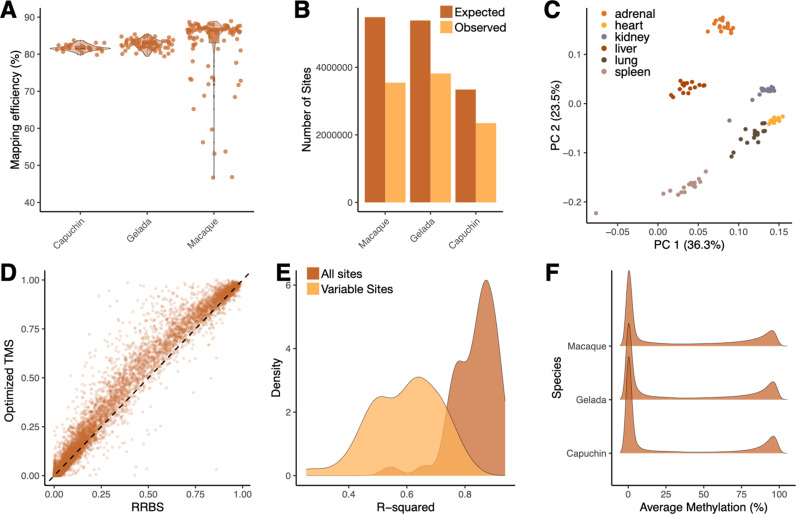
Optimized TMS performs well in non-human primate species and when compared to RRBS. **[A]** Optimized TMS in NHPs results in high mapping efficiencies despite the use of human-specific probes. Here, each of the species are mapped to their respective reference genome. We hypothesize that low mapping efficiency in certain rhesus macaque samples is due to variation in sample quality. **[B]** Number of expected and observed CpG sites covered in each NHP genome. Expected sites were derived from mapping the Twist probes to each NHP genome, while observed sites represent those detected with a coverage > 5X in >75% of samples. **[C]** Principal components analysis of TMS-derived DNA methylation levels for rhesus macaque samples spanning six distinct tissues. **[D]** Similar per CpG DNA methylation level estimates using RRBS (x-axis) and optimized TMS (y-axis) (R^2^ = 0.97). **[E]** Density plot of linear model R^2^ values obtained from comparing data generated via optimized TMS and RRBS for the same rhesus macaque samples. R^2^ values are provided when all CpG sites common to both technologies are included, as well as when only variably methylated (methylation > 10% and methylation < 90%) CpG sites are included. **[F]** Density curves of the average genome-wide DNA methylation level estimates for each NHP species. Curves show the expected bimodal distribution in which many of the CpG sites in the genome are either hypomethylated or hypermethylated.

To enable epigenomic analyses in our close primate relatives, we also tested whether TMS (96-plex, 200ng input protocol from experiment 1) could be effectively applied to three NHP species: tufted capuchins (*Sapajus apella*; n = 28 samples from blood), rhesus macaques (*Macaca mulatta*; n = 96 samples from 6 tissues (see S11 Fig and S16 Table)), and geladas (*Theropithecus gelada*; n = 68 samples from blood). While the probe set is designed from the human genome, NHP species share high levels of sequence homology with humans, especially in coding regions and regions near genes [61], leading us to hypothesize that a majority of CpG sites would be recovered. We mapped the Twist probe sequences to each of the NHP genomes to confirm this intuition, and from this analysis expected to capture 3.0-4.8 million CpG sites across the three species (Fig 4B). Importantly, for the rhesus macaque samples, we also generated paired RRBS data and compared our TMS results to a technology that does not rely on hybrid capture.

When examining initial quality control metrics, we found that all three NHP species had high mapping efficiencies (average = 81.96% for capuchins, 82.62% for geladas, and 81.35% for macaques; Fig 4A). Further, the average CHH methylation levels were all extremely low (<1%), again suggesting high conversion rates (S12 Fig). Following filtering, we recovered ~ ½ to ¾ of expected CpG sites in the NHP datasets (3,343,133 in capuchin, 5,387,280 in gelada, and 5,486,073 in macaque). The number of sites recovered scales generally with divergence time (capuchins share a common ancestor with humans 35–45 million years ago, geladas and rhesus macaques share a common ancestor with humans 23–28 million years ago) [62]. In all species, we were able to reliably measure more sites than would be typical of RRBS (see below), and we note that some of the between-species variation in performance could be explained by heterogenous read depth (S2 Table) as well as reference assembly quality. In particular, the quality of the rhesus macaque genome is much higher than the gelada or capuchin (using CNEr in R and the N50() and N90() commands [63]): mmul_10 N50 = 153,388,924, N90 = 79,627,064; tgel1 N50 = 147,341,205, N90 = 77,542,005; cimit N50 = 5,274,112, N90 = 1,283,179.

When examining average DNA methylation levels across species, we found that, as expected, all exhibited bimodal genome-wide profiles similar to humans (Fig 4F). Further, because the rhesus macaque samples were derived from 6 different tissue types (S11 Fig and S16 Table), we also confirmed that samples displayed expected tissue-specific epigenetic patterns. Specifically, we demonstrated that a Principal Components Analysis (PCA) was able to reliably separate samples by tissue type (Fig 4C), as has been observed in previous studies using both bisulfite sequencing and the EPIC array [64–66].

#### Experiment 8: Comparison of TMS to RRBS.

Studies of NHP species have historically relied on RRBS because of the species-specificity of microarray technologies and the cost barrier of WGBS [49,67,68]. To test how our optimized TMS protocol compares to RRBS, we generated paired data for all 96 rhesus macaque samples. After filtering both datasets to 721,766 common CpG sites, we found a high concordance of the average DNA methylation levels estimated by both technologies (R^2^ = 0.97; Figs 4D and S13). This remained true when we subsetted specifically to 92,692 variably methylated CpG sites (i.e., sites with average DNA methylation levels >0.1 and <0.9; R^2^ = 0.5945; Fig 4E).

### Biological analyses performed with TMS, EPIC, and RRBS data reveal similar results

#### Experiment 9: Epigenetic age and tissue-dependent patterns compared across technologies.

Thus far we have compared DNA methylation levels measured with TMS versus other technologies; if these measurements are robust across platforms, then power to detect biological patterns should also be similar. We thus asked whether data generated from paired samples, but using different technologies, could recapitulate 1) epigenetic age predictions using DNA methylation-based clock algorithms [7,69–73] and 2) tissue-dependent methylation signatures when comparing diverse organ systems. For analysis #1, we used the 55 VUMC cohort samples with paired EPIC and TMS data (focusing on 682,295 CpG sites passing filters and common to both technologies). For analysis #2, we used the 96 rhesus macaque samples with paired RRBS and TMS data (focusing on 391,758 CpG sites passing filters and common to both technologies).

**Fig 5:**
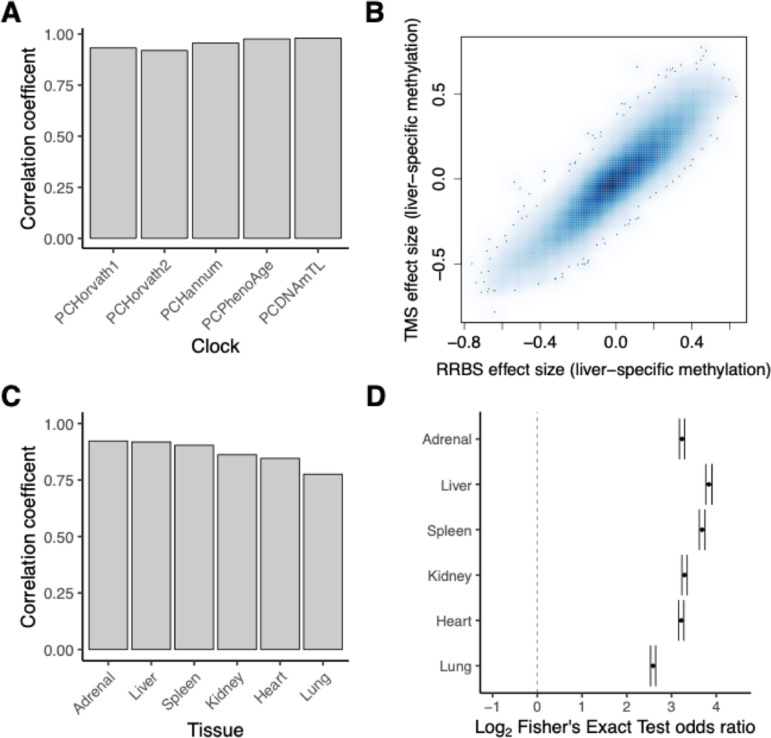
TMS recapitulates epigenetic age predictions and tissue-dependent effects identified via other technologies. **[A]** Pearson’s correlation coefficient comparing epigenetic age predictions for five PC-based epigenetic clocks run on TMS versus EPIC v2 array data from the VUMC cohort (n paired samples = 55). All correlations were significant following multiple hypothesis testing (FDR < 5%). **[B]** Correlation between standardized effect sizes, estimating liver-specific effects, using RRBS versus TMS data (n paired rhesus macaque samples = 96). To derive effect size estimates, models were run comparing the liver to all other tissues. Each point represents the effect size for a given CpG site common to both datasets. **[C]** Pearson’s correlation coefficient comparing effect sizes for estimates of tissue-specific effects using TMS versus RRBS data (n paired rhesus macaque samples per tissue = 96). Separate models were run for each tissue, comparing the focal tissue on the x-axis to all other other tissues to identify tissue-specific effects. All correlations were significant following multiple hypothesis testing (FDR < 5%). **[D]** Degree of enrichment (represented as an log2 odds ratio from a Fisher’s Exact test), between CpG sites identified as tissue-specific in TMS versus RRBS data using matched samples. Dashed line represents no enrichment and error bars represent confidence intervals.

For analysis #1, we observed a high correlation between epigenetic age estimates derived from TMS and EPIC data (Fig 5A; average R^2^ = 0.91). This agreement was high across a variety of existing epigenetic clock algorithms. For analysis #2, we found that effect size estimates of tissue dependency (for example, from linear models comparing DNA methylation levels in liver to all other tissues) were very similar genome-wide when applied to TMS versus RRBS data (Fig 5B and 5C). Additionally, we confirmed that sites identified as significantly associated (FDR < 5%) with a given tissue in RRBS versus TMS data overlapped more than expected by chance (Fig 5D). Together, these results support the ability of TMS data to uncover biological patterns in similar ways as other technologies.

## Discussion

We developed and benchmarked a multiplexed, cost-effective version of the TMS protocol and applied it to human populations from the US, Bolivia, and Malaysia as well as multiple NHP species. We recommend an optimal protocol for future work (96-plex, 200ng input, 65°C annealing, no methylation enhancer, enzymatic fragmentation), but found that data quality remained high across plexing strategies, input amounts, and protocol modifications. Importantly, the 96-plex version of the TMS protocol—including sequencing to achieve ~ 25x coverage per CpG site on the Illumina NovaSeq X—can currently be performed for ~USD 80 per sample (with roughly half being reagents and labor, and the other half being sequencing on the NovaSeq X platform; S1 Table). Relative to the commonly used EPIC array for human studies, this represents massive savings enabling larger-scale, population-based studies. We recognize that the total cost of TMS will vary by the amount of sequencing performed, and we provide simulations (and modifiable code) based on real TMS coverage distributions for users to explore the impact of coverage on power for a given study design. For example, we find that with a sample size of n = 100, moderate differences in methylation (e.g., 20%) can be identified with high power at relatively low read depths (e.g., 20x), while detecting small differences would require higher read depths. However, the relative impact of coverage on power diminishes at higher sample sizes. Researchers will thus need to tailor their sequencing plan based on both their expected effect size and the number of samples in hand (S3 Fig).

We found high concordance between TMS-derived DNA methylation levels and those derived from other commonly used methods—namely the EPIC array, WGBS, and RRBS. WGBS is the gold standard for comprehensive DNA methylation measurement, but is prohibitively expensive for most studies given the breadth of sequencing (to cover the whole genome) and the necessity for deep sequencing (to achieve high levels of precision) [50]. RRBS has filled in as a more cost-effective alternative, but due to the stochastic nature of the Msp1 digestion followed by size selection, not all CpG sites are reliably covered across individuals and missing data can impede downstream analyses (S14 Fig). We note that variation in coverage (and thus precision) across CpG sites will be an issue, to some degree, for any sequencing-based technology. As a result of these challenges, microarray-based methods have become the most commonly used approach in human genomics. Consequently, many popular bioinformatics pipelines and specialized algorithms for DNA methylation data (e.g., epigenetic clocks or cell type deconvolution [74,75]) are currently keyed to microarrays. While DNA methylation levels derived from TMS are strongly correlated with the EPIC array, it is important to keep in mind that: 1) a small subset of sites are not covered by both technologies, and 2) because microarrays output beta values (equivalent to methylated signal/(total signal + an offset)), the relationship between TMS- and EPIC-derived values cannot be exactly 1:1. We caution that care will thus be needed when applying existing microarray-based algorithms to TMS data, though our initial attempts at doing so with epigenetic clock algorithms do seem to perform well.

The study of DNA methylation in NHP species is deeply important to our understanding of gene regulatory evolution [76–78], comparative aging [67,68,79,80], and environmental impacts on phenotype [68,81]. For example, both captive and field-based NHP studies have strongly contributed to our understanding of how social and ecological inputs influence fitness-related traits through changes in DNA methylation [82,83]. These studies have sometimes relied on microarrays (e.g., the Mammalian Methylation Array [36,84–86] or the application of human arrays to NHP species [87–89]). However, given the high costs of arrays, a large proportion of previous work has relied on RRBS [68,80,81,83]. Although RRBS is easily adapted for non-human species, TMS can work with smaller amounts of input DNA than bisulfite-based protocols [42], which can be critical for studies of wild or endangered species. While TMS uses capture probes designed from the human genome, NHPs share high levels of sequence similarity, which we show is sufficient to reliably capture 2–3 million CpG. Though not all ~ 4 million CpG sites are captured, TMS still represents a consistent and cost-effective approach relative to the alternatives. Notably, we find that TMS is effective in both catarrhine (monkeys of Africa and Asia) and platyrrhine (monkeys of Central and South America) species, suggesting it may be effective in other members of these clades for capturing conserved regions. One potential issue that requires further study is that the probes (which are designed from human genetic variation) do not specifically avoid or take into account within-species polymorphisms.

To show that TMS data could detect expected biological patterns, in ways that are comparable to existing technologies, we performed the same analyses of epigenetic age estimation and tissue-specificity in matched TMS, EPIC, and RRBS data, respectively. From these analyses we found that the epigenetic ages estimated with TMS versus EPIC data were highly correlated as were genome-wide estimates of tissue specific patterns from TMS versus RRBS data. The potential portability of epigenetic clock algorithms is particularly encouraging, as this approach is becoming increasingly popular for measuring biological age [90–92], and will be exciting to pair with cost-effective methods going forward. Together, our optimized TMS protocol has the potential to add value and enable larger-scale studies in the many fields that query DNA methylation patterns, such as genetic medicine, developmental biology, evolutionary biology, anthropology, public health, geroscience, and more.

## Methods

### Ethics statement

For the Tsimane participants, informed consent was collected at three levels: by the individual (formal written consent), by the community, and by the Tsimane Gran Consejo (Tsimane governing body). All study protocols, including the generation of DNA methylation data, were approved by the Institutional Review Boards of the University of New Mexico (#07–157), the University of California Santa Barbara (#3-21-0652), and Universidad Mayor San Simon, Cochabomba.

For the Orang Asli participants, informed consent was also collected at multiple levels: first by first describing the project to the community as a whole and seeking the permission of community leaders, and subsequently through individual-specific review of the protocol and formal written consent. The study protocol, including the generation of DNA methylation data, was approved by Vanderbilt University (IRB #212175) as well as the Malaysian Medical Research Ethics Council.

For the rhesus macaque samples, the study protocol was approved by the Institutional Animal Care and Use Committee through the University of Puerto Rico’s Caribbean Primate Research Center (IACUC #A400117). For the gelada samples, the study protocol was approved by the Institutional Animal Care and Use Committees at the University of Washington (protocol 4416-01) and Arizona State University (20–1754 R) along with approval from the Ethiopian Wildlife and Conservation Agency. For the tufted capuchin samples, the study protocol was approved by the Institutional Animal Care and Use Committee at the Georgia State University (protocol A20018).

### Study populations, sample collection, and DNA extraction

Data generation drew on previously collected samples from multiple human and non human primate populations. A brief description of each population is provided below.

#### Tsimane of Bolivia.

The Tsimane are an Indigenous horticulturalists population spread across >90 villages in the Bolivian lowlands and totaling approximately 17,000 people [52]. We extracted DNA from 192 venous whole blood (WB) samples collected between the years of 2010–2021 as part of the Tsimane Health and Life History Project (THLHP). The THLHP has continuously collected demographic, behavioral, environmental, and health data along with the provision of medical services for over two decades [93]. Samples were frozen in liquid nitrogen, transferred on dry ice to Arizona State University, and stored at -80°C prior to analysis. The sample set for this project included 103 females and 89 males, with a mean age of 54.3 years old (range 18.0–83.6 years old) (see Table 1). Genomic DNA was extracted using the Zymo *Quick*-DNA 96 kit (Zymo Research #D3012) following the manufacturer’s instructions.

#### Orang Asli of Peninsular Malaysia.

The Orang Asli consist of ~19 ethnolinguistic groups and a total population of ~210,000 people [58]. They traditionally subsist on a mixture of hunting, gathering, fishing, small-scale farming, and trade of forest products [94,95]. We extracted DNA from 88 white blood cell (WBC) samples that were collected in 2023 as part of the Orang Asli Health and Lifeways Project (OA HeLP) [58]. Samples included in data generation were derived from venous blood draws followed by washing with QIAGEN PureGene red blood cell lysis. Samples were stored in liquid nitrogen upon collection, and at -80C for longer term storage. The Orang Asli sample included 46 females and 42 males, with a mean age of 35.3 years old (range 18–78 years old; Table 1). Genomic DNA was extracted using the Zymo *Quick*-DNA/RNA MagBead kit (Zymo Research #R2131) following the manufacturer’s instructions.

#### Vanderbilt University Medical Center cohort.

We were granted access to de-identified EPIC array data (Infinium MethylationEPIC v2.0 Kit) and TMS data from 55 paired human whole blood samples. These samples were sourced from a healthy cohort recruited through the Vanderbilt University Medical Center (VUMC) in Nashville, TN USA. Due to IRB restrictions, demographic data or other metadata were not available for these samples.

#### Rhesus macaques.

We obtained extracted DNA from rhesus macaque tissue samples (n = 96) collected by the Cayo Biobank Research Unit in partnership with the University of Puerto Rico’s Caribbean Primate Research Center (CPRC) [96–100]. Beginning in 2016, samples were collected from individuals living on the island of Cayo Santiago, an NIH-managed free-range colony of provisioned rhesus macaques. Specifically, as part of an ongoing population management plan designed by CPRC, select individuals were culled and tissues from all major organ systems were systematically harvested, stored in a fixative buffer, and frozen at -80C. This data set consists of samples from six different tissue types: adrenal, heart, kidney, lung, liver, and spleen, with 16 samples from each type and samples coming from 23 unique individuals (S3 Table). This dataset includes samples from 11 females and 12 males, ages 3.2 to 19.9 years old (mean 10.6 years old), collected from 2016–2019 (Tables 1 and S3). Genomic DNA was extracted using the Zymo *Quick*-DNA/RNA MagBead kit (Zymo Research #R2131) following the provided manufacturer’s protocols.

#### Geladas.

We extracted DNA from whole blood from 68 geladas; 21 were female and 47 were male and all were considered adult (i.e., over 4 years old, the minimum average age of reproductive maturation in this species [101]) (Table 1). Gelada samples were collected as part of the Simien Mountains Gelada Research Project (SMGRP) which, since 2017, has carried out annual capture-and-release campaigns to collect morphometric data and whole blood samples from wild Ethiopian geladas [102]. Samples were stored in liquid nitrogen upon collection, and at -80C for longer term storage. Genomic DNA was extracted using the Qiagen DNeasy Blood & Tissue kits (Qiagen #69581) following the provided protocols.

#### Tufted capuchins.

Blood was collected from individuals in the captive tufted capuchin monkey colony at Georgia State University in January 2023. Of the 28 capuchins, 19 were female and 9 were male with an average age of 19.4 years old (range 9–41 years old; Table 1). A trained veterinarian anesthetized the monkeys using 13 mg/kg Ketamine, delivered intramuscularly. Whole blood samples were collected during the monkeys’ annual physicals, stored at 4°C upon collection, and shipped to Arizona State University where they were flash frozen into 0.5mL aliquots and stored at -80°C until used for analysis. DNA was extracted using the Qiagen DNeasy Blood & Tissue kits (Qiagen #69581) following the manufacturer’s protocols.

### Overview of TMS library preparation

We used the Qubit dsDNA assay to determine the quantity of all extracted DNA. DNA libraries were normalized and prepared using the NEBNext Enzymatic Methyl-seq kit (P/N: E7120L) following a modified version of the manufacturer’s protocol that included 9 cycles of PCR for the final library amplification followed by a 0.65X bead cleanup. To prepare for the hybrid capture reaction, the total DNA input requirement (2000ng in this case) was divided by the total number of samples being pooled (12, 24, 48, or 96 as will be discussed below). In the 96-plex experiment, for example, 84ng of DNA from each sample was pooled totaling 8ug, and ¼ of the volume was used for the hybrid reaction and captured using the Human Methylome panel from Twist Biosciences following the manufacturer’s instructions (P/N: 105521). The final post-capture PCR reaction was split into 2 reactions per pool and cleaned with a 1X bead cleanup and then combined. Pool quality was assessed post-hybridization using the Agilent Bioanalyzer and quantified using a qPCR-based method with the KAPA Library Quantification Kit (P/N: KK4873) and the QuantStudio 12K instrument.

Prepared library pools were sequenced on the NovaSeq 6000 at the Vanderbilt Technologies for Advanced Genomics (VANTAGE) Core. We used 150 bp paired-end sequencing and generally targeted 30-50M paired-end reads per sample. Real Time Analysis Software (RTA) and NovaSeq Control Software (NCS) (1.8.0; Illumina) were used for base calling. MultiQC (v1.7; Illumina) was used for data quality assessments. For each sample, we applied the Illumina DRAGEN Methylation Pipeline v4.1.23 using the custom bed file from Twist Biosciences. The deliverables from DRAGEN consist of FASTQs, bams, cytosine reports (which include counts of methylated and unmethylated reads per CpG site), and methyl and mapping metric reports.

### TMS library preparation for experiments 1 & 2: Varying multiplexing strategies and input amounts

To determine whether TMS can be effectively multiplexed beyond the recommended 8-plex, we used 96 Tsimane samples to test four different multiplexing strategies during capture: 12-, 24-, 48-, and 96-plex. To test whether TMS is robust to DNA input amounts, we tested five input amounts: specifically, 25, 50, 100, 200, and 400 ng of sample were used as input into the EM-seq library prep. Here, we kept the plexing strategy constant (12-plex) and used three Tsimane samples, each represented three times within each pool and included three replicates of a control sample (HG01879 from the 1000 Genomes Project) [103].

### TMS library preparation for experiments 3 & 4: Optimizing capture efficiency and DNA fragmentation strategies

To optimize the capture efficiency of Twist target sites, we tested the use of two different annealing temperatures–65° and 68° C–along with the use of a methylation enhancer (ME)– produced by Twist Biosciences (Catalog #103557) consisting of Tris EDTA buffer to block the binding of off-target probes thereby improving on-target capture efficiency. The specific combinations we explored were: testing a 65°C annealing temperature with 0uL (n = 192), 2uL (n = 96), and 4uL (n = 96) of ME and testing a 68°C annealing temperature with 0uL (n = 96) and 2uL (n = 192) of ME. These experiments were conducted with 96-plexed Tsimane samples (n = 192), and using 200 ng of sample input.

Next, we tested the use of an enzymatic fragmentation method to replace the Covaris (LE220) mechanical fragmentation approach. Mechanical fragmentation is known to decrease library quality through damage to DNA; however, enzymatic fragmentation is not currently recommended by the TMS protocol. To compare these approaches, we performed the optimized TMS with enzymatic fragmentation using 4uL of NEBNext UltraShear (NEB #M7634S/L) for 10 or 20 minutes. This experiment was conducted using 96-plexed samples from the Orang Asli (n = 88) and using 200 ng of sample input.

### TMS and RRBS library preparation for experiments 7 and 8

To evaluate the efficacy of optimized TMS on three NHP species—macaques, geladas, and capuchins—we applied the 96-plex protocol design from experiment 1 with 200 ng input. To compare rhesus macaque TMS to RRBS, we generated RRBS libraries using 150 ng of DNA input in combination with 1ng of lambda phage DNA and 1uL of Msp1—a digestive enzyme which cuts CCGG nucleotide motifs. Next, using NEBNext Ultra II reagents, we performed end repair and adapter ligation to the DNA fragments produced by Msp1 digestion. We then performed bisulfite conversion on the fragments using the EZ-96 DNA Methylation Lightning MagPrep kit (Zymo Research #D5046) following the manufacturer directions. The samples were then PCR amplified for 16 cycles with unique dual indexed sequencing primers. We selected for 180–2000 bp fragments and sequenced on an Illumina NovaSeq S2 flow cell with 2x51bp sequencing [80,104].

### Low-level processing of TMS data

For experiments 1, 2, 7, and 8, we used a custom bioinformatics pipeline to process all FASTQ files into counts of methylated versus unmethylated cytosines at each CpG site. For experiments 3, 4, 5, and 6, we used Illumina’s Dynamic Read Analysis for GENomics (DRAGEN) pipeline [105] to process all FASTQ files into counts of methylated versus unmethylated cytosines at each CpG site. Importantly, both our custom pipeline and DRAGEN follow the same general steps and rely on the Bismark suite [106], making them highly comparable. We also processed 7 samples from experiment 4 using both methods to empirically confirm that our custom pipeline and the Illumina DRAGEN pipeline produced near identical results (S15 Fig).

For our custom pipeline, we first trimmed adapters using Trimmomatic (version 0.39) [107] and TrimGalore (version 0.6.6) [108] for human and NHP samples, respectively. Following trimming, we used Bismark (version 0.24.0) [106] to map reads to each species’ respective genomes (hg38 for human, mmul10 for rhesus macaque, cimit for capuchin, and tgel1 for gelada). We retained only uniquely mapped reads and used the methylation extractor function within Bismark to extract counts of methylated versus unmethylated cytosines at each cytosine. These files were further filtered for CpG contexts only.

For all samples, run through either the custom or DRAGEN pipeline, we extracted two measures of data quality that are automatically calculated by Bismark: the percent of reads that mapped uniquely to the reference genome and the average methylation percentage for cytosines in a CHH context. The latter value serves as a commonly used estimate of the efficiency with which a given protocol converts unmethylated cytosines to thymine, because cytosines located outside of CpG contexts are extremely unlikely to be methylated in the mammalian genome [109,110]. Estimates of CHH methylation were extracted from an automatically generated report file when using Bismark to align the trimmed FASTQ files to the reference genome. For experiments 1 and 2, we tested whether multiplexing strategy and input amount impacted mapping efficiency and percent CHH methylation using a one-way ANOVA test, followed by a pairwise t-test in the case of significance, with the ‘aov’ and ‘pairwise.t.test’ functions in the ‘stats’ R package [111].

For each study, we used the BSseq R package [112] to compile count matrices (derived from our custom pipeline or DRAGEN) across samples and to perform region, coverage, and missingness filtering. For experiments 1, 3, 4, 5, and 6 we used built-in functions in BSseq to filter for sites within the probes regions (+/- 200 bp) and for sites covered at>5X in >75% of samples. We made slight modifications to this filtering pipeline for other experiments. For experiment 2, where n = 3 for each input amount, we relaxed our missingness filter to sites with at least one read observed in at least ⅔ samples. For experiment 7, which focused on NHP genomes for which the probe set coordinates (which are provided in hg38) are irrelevant, we did not perform region filtering. The number of sites analyzed for each experiment (reported in the main text and in S4 Fig) therefore varies slightly depending on sample size, sequencing coverage, and other factors that impact which CpG sites passed our filters.

To confirm the fidelity of optimized TMS, we also checked whether CpGs captured by the protocol were distributed as expected throughout different genomic regions (e.g., promoters, enhancers) and that the average methylation levels in different genomic regions were as expected. To do so, we annotated each CpG site by whether it fell into a gene body, promoter, or non-genic region, and by chromatin state. We used hg38 gene body coordinates from Ensembl’s ‘biomaRt’ package in R, and we defined promoter regions as the 2000 bp region upstream of TSSs. We annotated CpGs as falling in chromatin states as defined by hg38 ChromHMM annotations from NIH’s Roadmap Epigenomics Project [54]. We then counted the number of CpG sites that fell in each region (Fig 2C) and evaluated the median methylation across samples (Fig 2D).

### Quantifying capture efficiency

A subset of our experiments sought to understand and optimize two measures of efficiency of the hybrid capture step: 1) how many of the expected CpG sites from the probe set passed our filtering parameters and were thus analyzable and 2) how many of the reads we generated for a given sample were on-target and putatively captured by the probe set, rather than representing off-target randomly sequenced DNA fragments that do not contribute to analyzable data as they are often sparsely shared between samples. For #1, we used the bedtools (version 2.28.0) [113] intersect command to determine the proportion of CpG sites that are within +/- 200 bp with at least 1 probe [using a bed file available on the Twist Biosciences website (https://www.twistbioscience.com/resources/data-files/twist-human-methylome-panel-target-bed-file)]. For #2, we used the bedtools function bamtobed to convert the mapped reads for each sample into a bed file; because we used a paired end sequencing strategy, each bed coordinate included a fragment start position from R1 and a fragment end position from R2. We then used the bedtools intersect command to determine the proportion of mapped read pairs that are within 200 bp of at least 1 Twist probe.

### Simulating TMS data to estimate power across coverages, effect sizes, and sample sizes

To understand what level of coverage is necessary to detect particular effect sizes in different sample sizes, we conducted a power analysis using simulated data based on the true coverage distribution of 1,000 sites in our TMS dataset, drawing with replacement to simulate sample sizes of n = 100, 200 and 400 (following the methods in [49,59]). We then assigned each sample a binary predictor variable (0 or 1), estimated methylation level differences between groups for a given effect size, and simulated the number of methylated counts per sample we would observe under this scenario by sampling from a binomial distribution (given the number of total counts and the probability of a count being methylated). We simulated data for effect sizes ranging from a 0–20% difference in methylation levels between groups and calculated power as the proportion of sites in which the predictor variable had a significant effect on methylation at a nominal p-value threshold of 0.001. We ran this analysis 3 times, first using the true mean coverage of our dataset (~20x), then again simulating coverage of 40x and 80x by multiplying the total counts of each site by 2 and 4, respectively.

### Comparing DNA methylation measurements between TMS, the EPIC array, and WGBS

We used our filtered BSSeq object from experiment 5 to compare to data from the EPIC array generated for 55 paired human samples (average number of CpG sites measured with EPIC = 936,280; average call rate = 0.999). We downloaded the EPIC CpG coordinates from the Illumina website and merged with the TMS CpG locations, resulting in a shared dataset of 682,295 CpG sites passing filters and common to both technologies. We then performed two analyses to understand consistency. First, we calculated the average per-site methylation level across all samples included in the TMS or EPIC array datasets, respectively. We then ran a linear model testing the relationship between the two sets of average methylation levels using the ‘lm’ function in the ‘stats’ package in R. Second, we used the ‘lm’ function to estimate the R^2^ value comparing per-site methylation levels for estimates derived from each technology for a given individual (i.e., not averaged across the dataset). This resulted in a distribution of 55 R^2^ values. Because all humans share canonical methylation patterns, we also compared this distribution to a distribution of 55 R^2^ values derived from the same analysis after sample identity was permuted. We used the ‘t.test’ function in the ‘stats’ package in R to ask whether these distributions were significantly different.

We used a very similar strategy to compare ~ 30x WGBS data generated for six paired Tsimane samples with TMS data generated from experiment 1 (96-plex, 200 ng input). First, we performed low level processing of the WGBS data using Illumina’s DRAGEN pipeline and merged this with our filtered TMS data, resulting in 3,078,771 CpG sites common to both datasets. We calculated the average methylation level across samples reported for each site and technology and ran a linear model using the ‘lm’ function in the ‘stats’ package in R to calculate the R^2^ value. We did not compare individual-based R^2^ values to permuted values for this experiment, given the small number of individuals.

### Understanding TMS performance in NHP species and comparing DNA methylation measurements between TMS and RRBS

To estimate the number of CpG sites that we expected to recover when applying the human probe set to each NHP species, we converted the probe bed file to a FASTA file using the bedtools command ‘getfasta’ [113] and the hg38 reference genome. We then used Bismark to map the FASTA file to each non-human primate’s respective genome. From the mapped bam file, we used the ‘bamToBed’ function in bedtools to extract coordinates for the mapped probes and to add a + /- 200 bp offset. Finally, we applied the ‘getfasta’ function in bedtools to extract the sequence for the mapped regions (plus the 200 bp buffer) from the non-human primate genome and to count the number of CpG sites in this region set.

Similar to the comparisons between TMS and the EPIC array, we used paired RRBS data for the 96 rhesus macaque samples to directly compare methylation data generated using TMS versus RRBS. To do so, we processed the RRBS data using the same custom pipeline and filtering parameters described for TMS data, with the only modification being that we used the ‘—rrbs’ parameter in TrimGalore to remove unmethylated cytosines artificially introduced during library preparation from the 3’ end of fragments. We merged the filtered TMS and RRBS datasets, resulting in 721,766 CpG sites common to both technologies. As described for the TMS-EPIC array comparison, we then 1) calculated the average per-site methylation level across all samples included in each dataset and compared these vectors using linear models and 2) estimated the R^2^ value for methylation level estimates derived from each technology for a given individual, and used a t-test to compare this distribution to a distribution for the same analysis where sample identity was permuted (S16 Fig).

### Testing for tissue-specific DNA methylation patterns and estimating epigenetic age using data from different technologies

First, we compared epigenetic age predictions from paired samples that were sequenced on different platforms. We estimated epigenetic age from the PC-based versions of five well-established epigenetic clocks, including the Horvath multi-tissue clock, Hannum blood clock, PhenoAge clock, and telomere length clock. The PC-based versions of these clocks have much higher reliability and less susceptibility to technical noise than the original CpG-site level clocks [73]. We estimated epigenetic age from these clocks using the PC-Clocks R package [114] and calculated the Pearson’s correlation coefficient for estimates from samples generated with TMS versus the EPIC array.

Second, we compared tissue-specific effect size estimates between samples generated with RRBS and TMS. Specifically, we asked whether tissue type significantly (FDR < 5%) predicted DNA methylation among the multi-tissue macaque data for each technology, using beta binomial models implemented in the R package ‘aod’. We performed these analyses iteratively to compare a given tissue to all other tissues (for example, comparing liver versus all other tissues to estimate liver-specific effects). We limited this analysis to variably methylated CpG sites (median methylation <90% or >10%).

## Supporting information

S1 Table Itemized cost-per-sample breakdown of TMS. (XLSX)

S2 Table Read depth and metadata per sample, broken down by experiment and condition. (XLSX)

S3 Table Comparison of mapping efficiency with varying DNA input amounts. P-values generated from pairwise t-tests comparing the percentage of reads that were uniquely mapped to the human genome from sequencing data generated using the TMS protocol with varying amounts of input DNA. * represents a significant (p < 0.05) difference in mapping efficiency between conditions.
(XLSX)

S4 Table Comparison of CHH methylation with varying plexing strategies. P-values generated from pairwise t-tests comparing the percentage of cytosines in a CHH context marked as methylated (an estimate of conversion efficiency). * represents a significant (p < 0.05) difference in percent CHH methylation between conditions.
(XLSX)

S5 Table Comparison of CHH methylation with varying input amounts. P-values generated from pairwise t-tests comparing the percentage of cytosines in a CHH context marked as methylated (an estimate of conversion efficiency). * represents a significant (p < 0.05) difference in percent CHH methylation between conditions.
(XLSX)

S6 Table Number of captured on-target sites and average site-based coverage for each plexing strategy. Sites are filtered for those within the Twist probe set and covered at>5x coverage in >75% of samples. The average number of reads is provided as the total, such that the number of paired end reads would be ½ the reported value. 200ng of DNA was used for each sample for all plexing experiments.
(XLSX)

S7 Table Number of captured on-target sites and average site-based coverage for each input amount. Sites are filtered for those within the Twist probe set and covered at>5x coverage in >75% of samples. The average number of reads is provided as the total, such that the number of paired end reads would be ½ the reported value. All input experiments were pooled using the 12-plex strategy.
(XLSX)

S8 Table Percent of probes represented for each plexing strategy. The percentage of Twist target probes (n = 551,803) covered by at least one read for each plexing strategy.
(XLSX)

S9 Table Percent of probes represented for each input amount. The percentage of Twist target probes (n = 551,803) covered by at least one read for each input amount.
(XLSX)

S10 Table Correlation in average methylation at each site between plexing strategies. R^2^ values generated using linear modeling to compare average site-level methylation between plexing experiments. Average site-level methylation was calculated by averaging the percent methylation for each site across all samples within a given plexing strategy and comparing these with average site-level methylation within shared sites in an alternate plexing strategy. All sites were filtered for>5X coverage in >75% of samples.
(XLSX)

S11 Table Correlation in average methylation at each site between input amounts. R^2^ values generated using linear modeling to compare average site-level methylation between input amount experiments. Average site-level methylation was calculated by averaging the percent methylation for each site across all samples within a given input amount experiment and comparing with average site-level methylation within shared sites in an alternate input amount experiment. All sites were filtered for>5X coverage in >75% of samples.
(XLSX)

S12 Table Comparison of percent off-target reads with varying protocol modifications. P-values generated from pairwise t-tests comparing the percentage of the total reads that were not associated with a Twist target probe (within +/- 200 bp) for each capture efficiency experiment. 65C/68C refers to annealing temperature and 0uL/2uL/4uL ME refers to volume of methylation enhancer. * represents a significant (p < 0.05) difference in the percent of probes captured between conditions.
(XLSX)

S13 Table Comparison of probe capture with varying protocol modifications. P-values generated from pairwise t-tests comparing the percentage of Twist target probes covered by at least one read for each capture efficiency experiment. 65C/68C refers to annealing temperature and 0uL/2uL/4uL ME refers to volume of methylation enhancer. * represents a significant (p < 0.05) difference in the percent of probes captured between conditions.
(XLSX)

S14 Table Number of captured on-target sites and average site-based coverage for each capture efficiency experiment. Sites are filtered for those which are covered at>5x coverage in >75% of samples.
(XLSX)

S15 Table Correlation in methylation across DNA fragmentation methods. R^2^ values generated using linear modeling to compare site-specific methylation for 3 samples, each processed with 3 different fragmentation methods- mechanical, enzymatic for 10 minutes, and enzymatic for 20 minutes. All sites were filtered for>5X coverage in >75% of samples.
(XLSX)

S16 Table Rhesus macaque multi-tissue dataset. Age, sex, and tissue types for each individual in the rhesus macaque multi-tissue dataset, used to assess the function of TMS in a NHP with a direct comparison to RRBS data generated from these same samples (see also S8 Fig).
(XLSX)

S1 Text Supplementary methods. (DOCX)

S1 Fig Comparison of CHH methylation across experiments. Percentage of cytosines in a CHH context marked as methylated (an estimate of conversion efficiency) for varying (A) plexing strategies, and (B) input amounts. The dashed line refers to 1% CHH methylation and the solid line refers to 5% CHH methylation, a common cut off indicative of high levels of unmethylated cytosine conversion.
(TIFF)

S2 Fig Distribution of average per-site coverage and average paired-end reads broken down by experiment. (A) Average coverage per CpG site passing filters in a given experiment. Prior to calculations, CpG sites were filtered to include only sites within 200 bp of target probes and those with>5X coverage in more than 75% of samples. (B) Average read depth, in terms of paired-end reads, generated per sample in each experiment.
(TIFF)

S3 Fig Relationship between effect size, coverage, sample size, and power. Power analyses conducted on data for 1,000 simulated CpG sites (per sample size, effect size, and coverage combination) using the coverage distributions of observed, 96-plex TMS data. Lines represent the power to detect a 0–20% difference in methylation between two groups at a nominal p-value threshold < 0.001. Colors represent different levels of mean coverage per site (20x, 40x, and 80x) and facets represent sample sizes of n = 100, n = 200, and n = 400.
(TIFF)

S4 Fig Number of on-target CpG sites represented in each experiment. Number of CpG sites within 200 bp of target probes after filtering for>5X coverage in more than 75% of samples by experiment. Colors are representative of each experiment which are defined in Fig 1C.
(TIFF)

S5 Fig Correlation in average site-level methylation for varying fragmentation methods. Site-level methylation averaged across 3 samples processed using mechanical fragmentation, enzymatic fragmentation for 10 minutes, and enzymatic fragmentation of 20 minutes. Each point represents a site measured across both fragmentation methods and R2 values were generated using linear modeling.
(TIFF)

S6 Fig Average and median coverage for on-target sites when mapped read files are subset to varying degrees. We subset the mapped read files for each sample (n = 88) included in our enzymatic fragmentation experiment (experiment 4) to include a random subset of 25, 50, or 75% of the total reads. We calculated the average (A) and median (B) coverage for on-target sites (y-axis) and observed a linear relationship between coverage and the number of subset reads (x -axis), which is useful for estimating what sequencing depth per sample will be needed to obtain various degrees of coverage.
(TIFF)

S7 Fig Correlation in average DNA methylation levels between TMS and EPIC data split by (A) EPIC v2 Type I and (B) Type II probes. DNA methylation levels (A: n = 115,982 matched CpG sites; B: n = 575,401 matched CpG sites) averaged across 55 VUMC samples processed using TMS and the EPIC v2 array. Each point represents a site measured across both processing methods and R2 values were generated using linear modeling.
(TIFF)

S8 Fig Correlation in average DNA methylation levels between TMS and EPIC data split by different subsets of the genome. For samples processed using both TMS and the EPIC array (n = 55 VUMC samples), we assessed the correlation in site-level average methylation levels for (A) hypomethylated regions (<50% average methylation); (B) intermediately methylated regions (average methylation above 10% and below 90%); (C) hypermethylated regions (>50% methylation); (D) UCSC-annotated CpG islands; (E) UCSC-annotated CpG shores (i.e., regions within 2kb of the boundaries of a CpG island); and (F) UCSC-annotated CpG shelves (regions within 2kb and 4kb of the boundaries of a CpG island R2 values were calculated using linear modeling; sample sizes represent the number of CpG sites included in each panel.
(TIFF)

S9 Fig Correlation in site-level methylation between TMS and EPIC array data after sample permutation. For samples processed using both TMS and the EPIC array, we assessed the correlation in site-level methylation for variable sites (methylation >0.1 and <0.9) and all sites after permuting sample ID randomly. R2 values were generated using linear modeling.
(TIFF)

S10 Fig Average methylation and coverage across technologies. (A) Density plot showing the average methylation of a site (i.e., across samples) for filtered (>5X coverage in >75% of sites) sites captured between the three technologies (726,597 EPIC Array sites; 4,990,351 TMS sites; and 5,000,659 WGBS sites). Sites were not matched between the three technologies. (B) Average coverage per site of sites captured by WGBS after filtering for>5X coverage in >75% of samples. Median average coverage is 24.0X.
(TIFF)

S11 Fig Number of individuals from which different numbers of tissues were included in the rhesus macaque multi-tissue dataset. The majority of individuals had 4 + tissues represented in the dataset.
(TIFF)

S12 Fig Comparison of CHH methylation across experiments testing optimized TMS in three NHP species. Percentage of cytosines in a CHH context marked as methylated (an estimate of conversion efficiency) following optimized TMS using genomic DNA from capuchins, geladas, and macaques. The dashed line refers to 1% CHH methylation and the solid line refers to 5% CHH methylation, a common cut off indicative of high levels of cytosine conversion.
(TIFF)

S13 Fig Correlation in average site-level DNA methylation estimates between TMS and RRBS. Site-level DNA methylation estimates averaged across 96 rhesus macaque samples processed using TMS and RRBS. Each point represents a site measured across both fragmentation methods and R2 values were generated using linear modeling. RRBS enriches for CpG dense regions of the genome, which tend to be hypomethylated.
(TIF)

S14 Fig Number of samples for which a site is covered across for datasets generated using (A) RRBS and (B) TMS. Sites filtered for>5X coverage in >75% of samples processed using a given technology. A greater number of sites are covered consistently across all 96 samples using TMS compared to RRBS.
(TIFF)

S15 Fig Correlation in average site-level methylation between samples processed using Illumina’s DRAGEN pipeline and our custom pipeline. Each point represents the average methylation at a given site for 88 samples that were processed using both pipelines (R2 = 0.9972).
(TIFF)

S16 Fig Correlation in site-level DNA methylation estimates between TMS and RRBS data after permutation. For samples processed using both TMS and RRBS, we assessed the correlation in site-level methylation for all sites after permuting sample ID randomly and compared them to non-permuted, or matched, sample IDs. R2 values were generated using linear modeling. Using a t.test, we found a significant difference between the means of the two samples (t = 7.6796, p-value = 8.224 x 10–13, mean of matched samples: 0.8345, mean of permuted samples: 0.7508).
(TIFF)
