# Supplementary material for: Cost-effective solutions for high-throughput enzymatic DNA methylation sequencing
Source: PLoS Genet. 2025 May 22;21(5):e1011667. doi: 10.1371/journal.pgen.1011667 (PMC12162101; doi:10.1371/journal.pgen.1011667)
Supplement: S1 Text — (DOCX) [file pgen.1011667.s017.docx]

Supplemental methods for Longtin et al.,

**“Cost-effective solutions for high-throughput enzymatic DNA methylation sequencing”**

**SUPPLEMENTAL METHODS**

## **Parameters used for low level data processing of TMS data**

For experiments 1, 2, 7, and 8, we used a custom bioinformatics pipeline to process all FASTQ files into counts of methylated versus unmethylated cytosines at each CpG site. We trimmed the FASTQ files from experiments 1 and 2 using Trimmomatic (ILLUMINACLIP:TruSeq3-PE.fa: 2:30:10:8:trueHEADCROP:3 TRAILING:10 MINLEN:25) (version 0.39) [(1)](https://paperpile.com/c/qyQjVL/Uo5z) and we trimmed the FASTQ files from experiments 7 and 8 using TrimGalore (--paired, -j 8) (version 0.6.6) [(2)](https://paperpile.com/c/qyQjVL/eLVM). Following trimming, we processed all files using Bismark (version 0.24.0) [(3)](https://paperpile.com/c/qyQjVL/t6ZU) for mapping (hg38 for human, mmul10 for macaque, cimit for capuchin, and tgel1 for gelada) (--score_min L,0,-0.6) and methylation extraction (bismark_methylation_extractor: -p, --bedGraph, --comprehensive; coverage2cytosine: --merge_CpG). For experiments 3, 4, 5, and 6, we downloaded cytosine reports (the equivalent files produced from Illumina’s DRAGEN pipeline, which relies on the Bismark suite [(4)](https://paperpile.com/c/qyQjVL/HEFS)) from Ilumina’s BaseSpace CLI interactive portal.

This Illumina’s DRAGEN pipeline as well as the in-house commands described above generate 1) a cov.gz file used for downstream analyses and 2) multiple report files containing information such as mapping efficiency and average CHH methylation level (which were extracted and summarized in the main text). We used the .cov.gz files and combined them within a given experiment using the BSseq package in R [(5)](https://paperpile.com/c/qyQjVL/2pDr) to generate an RDS objects using the read.bismark command (files = list.files, verbose = TRUE, strandCollapse = TRUE, backEnd = “HDF5Array”). For each experiment, this object was filtered (using built in functions in BSseq) to include CpG sites within the Twist probe set, that were covered at >5x on average across samples, and that had data for >75% of samples.

**Estimating the number of reads needed to achieve a given coverage**

We used data from experiment 4, which represents our “best” protocol (96-plex, 200ng input, 65ºC annealing, no methylation enhancer, enzymatic fragmentation), to understand a critical aspect of experimental design—how many reads one would need to generate to achieve a given average coverage per CpG site. To do so, we used the ‘view’ function (-@ 20 -bh -s) in SAMtools [(6)](https://paperpile.com/c/qyQjVL/rRNx) to subsample the uniquely mapped reads resulting from the enzymatic fragmentation experiment for each sample (specifically subsampling to include 25%, 50%, and 75% of the mapped reads in each bam file). We then reran our sample processing pipeline starting with the bam/mapped reads file (see previous section) and calculated the average coverage per CpG site across all sites represented by each original file or subsampled file. We then compared the number of uniquely mapped reads to this value. In general, we observe a 1:2 relationship between the number of mapped, paired end reads (in millions) and average coverage, such that ~25M mapped paired end reads translates to ~50x average coverage per CpG site. The results of this analysis can be found in Figure S5.

**REFERENCES**

1. [Bolger AM, Lohse M, Usadel B. Trimmomatic: a flexible trimmer for Illumina sequence data. Bioinformatics. 2014 Aug 1;30(15):2114–20.](http://paperpile.com/b/qyQjVL/Uo5z)

2. [Krueger F, James F, Ewels P, Afyounian E, Weinstein M, Schuster-Boeckler B, et al. FelixKrueger/TrimGalore: v0.6.10 - add default decompression path [Internet]. Zenodo; 2023. Available from:](http://paperpile.com/b/qyQjVL/eLVM) <https://zenodo.org/record/7598955>

3. [Krueger F, Andrews SR. Bismark: a flexible aligner and methylation caller for Bisulfite-Seq applications. Bioinformatics. 2011 Jun 1;27(11):1571–2.](http://paperpile.com/b/qyQjVL/t6ZU)

4. [Krueger F, Andrews SR. Bismark: a flexible aligner and methylation caller for Bisulfite-Seq applications. Bioinformatics. 2011 Jun 1;27(11):1571–2.](http://paperpile.com/b/qyQjVL/HEFS)

5. [Park Y, Wu H. Differential methylation analysis for BS-seq data under general experimental design. Bioinformatics. 2016 May 15;32(10):1446–53.](http://paperpile.com/b/qyQjVL/2pDr)

6. [Li H, Handsaker B, Wysoker A, Fennell T, Ruan J, Homer N, et al. The Sequence Alignment/Map format and SAMtools. Bioinformatics. 2009 Aug 15;25(16):2078–9.](http://paperpile.com/b/qyQjVL/rRNx)
