# Supplementary figures and images for: Cost-effective solutions for high-throughput enzymatic DNA methylation sequencing
Source: PLoS Genet. 2025 May 22;21(5):e1011667. doi: 10.1371/journal.pgen.1011667 (PMC12162101; doi:10.1371/journal.pgen.1011667)

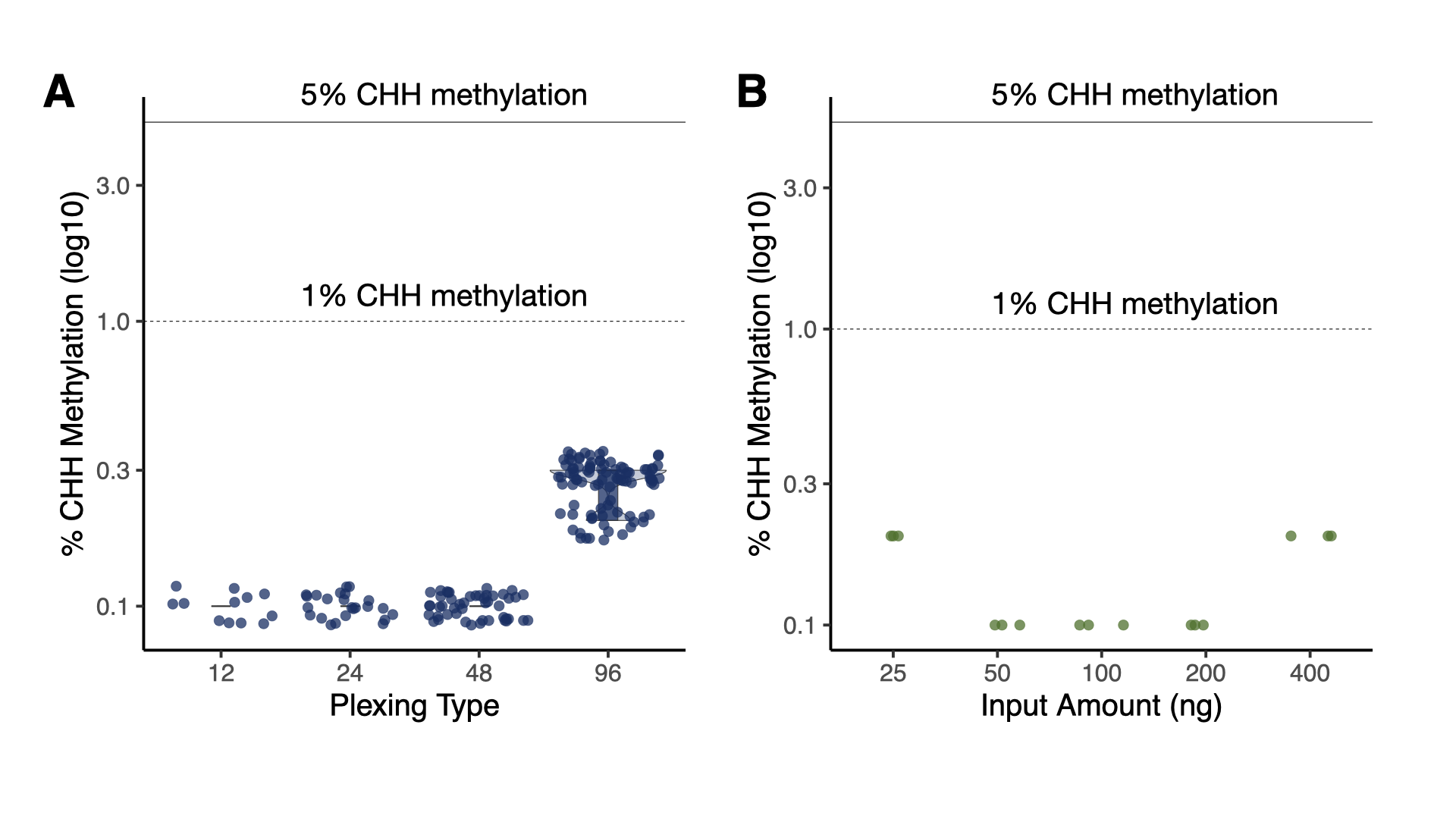

Supplement: S1 Fig — Percentage of cytosines in a CHH context marked as methylated (an estimate of conversion efficiency) for varying (A) plexing strategies, and (B) input amounts. The dashed line refers to 1% CHH methylation and the solid line refers to 5% CHH methylation, a common cut off indicative of high levels of unmethylated cytosine conversion. (TIFF) [file pgen.1011667.s018.tiff]

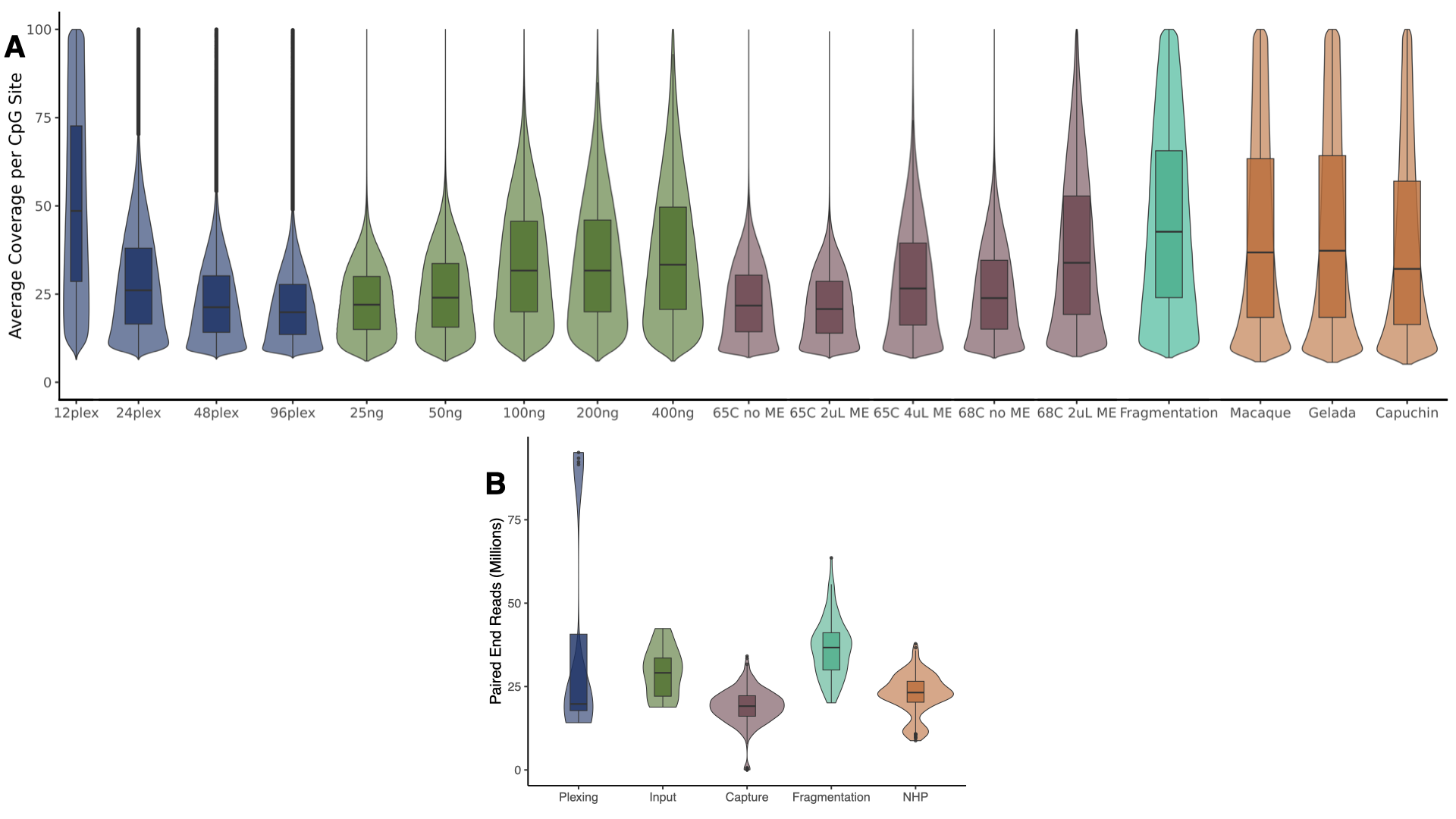

Supplement: S2 Fig — (A) Average coverage per CpG site passing filters in a given experiment. Prior to calculations, CpG sites were filtered to include only sites within 200 bp of target probes and those with>5X coverage in more than 75% of samples. (B) Average read depth, in terms of paired-end reads, generated per sample in each experiment. (TIFF) [file pgen.1011667.s019.tiff]

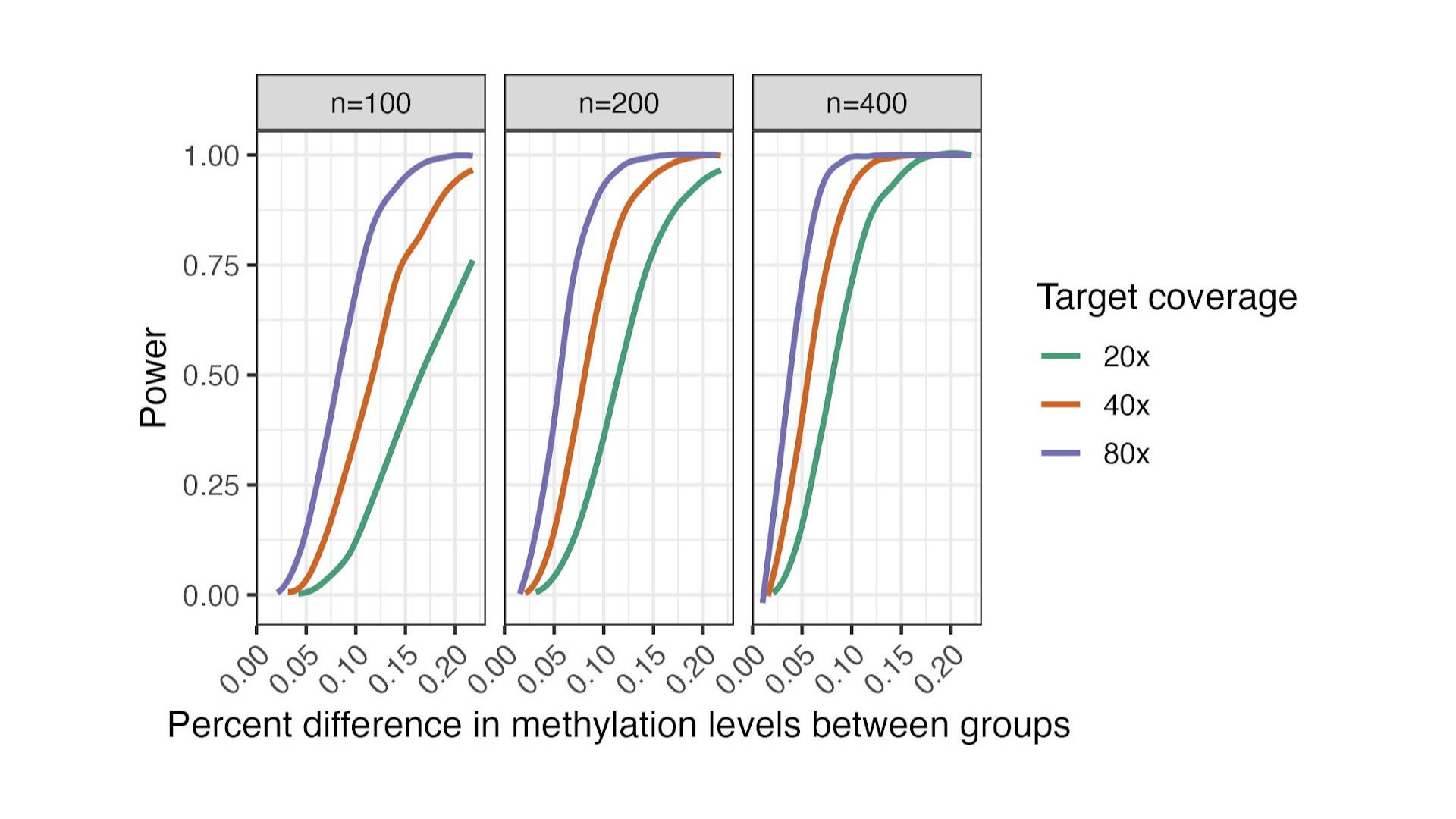

Supplement: S3 Fig — Power analyses conducted on data for 1,000 simulated CpG sites (per sample size, effect size, and coverage combination) using the coverage distributions of observed, 96-plex TMS data. Lines represent the power to detect a 0–20% difference in methylation between two groups at a nominal p-value threshold < 0.001. Colors represent different levels of mean coverage per site (20x, 40x, and 80x) and facets represent sample sizes of n = 100, n = 200, and n = 400. (TIFF) [file pgen.1011667.s020.tiff]

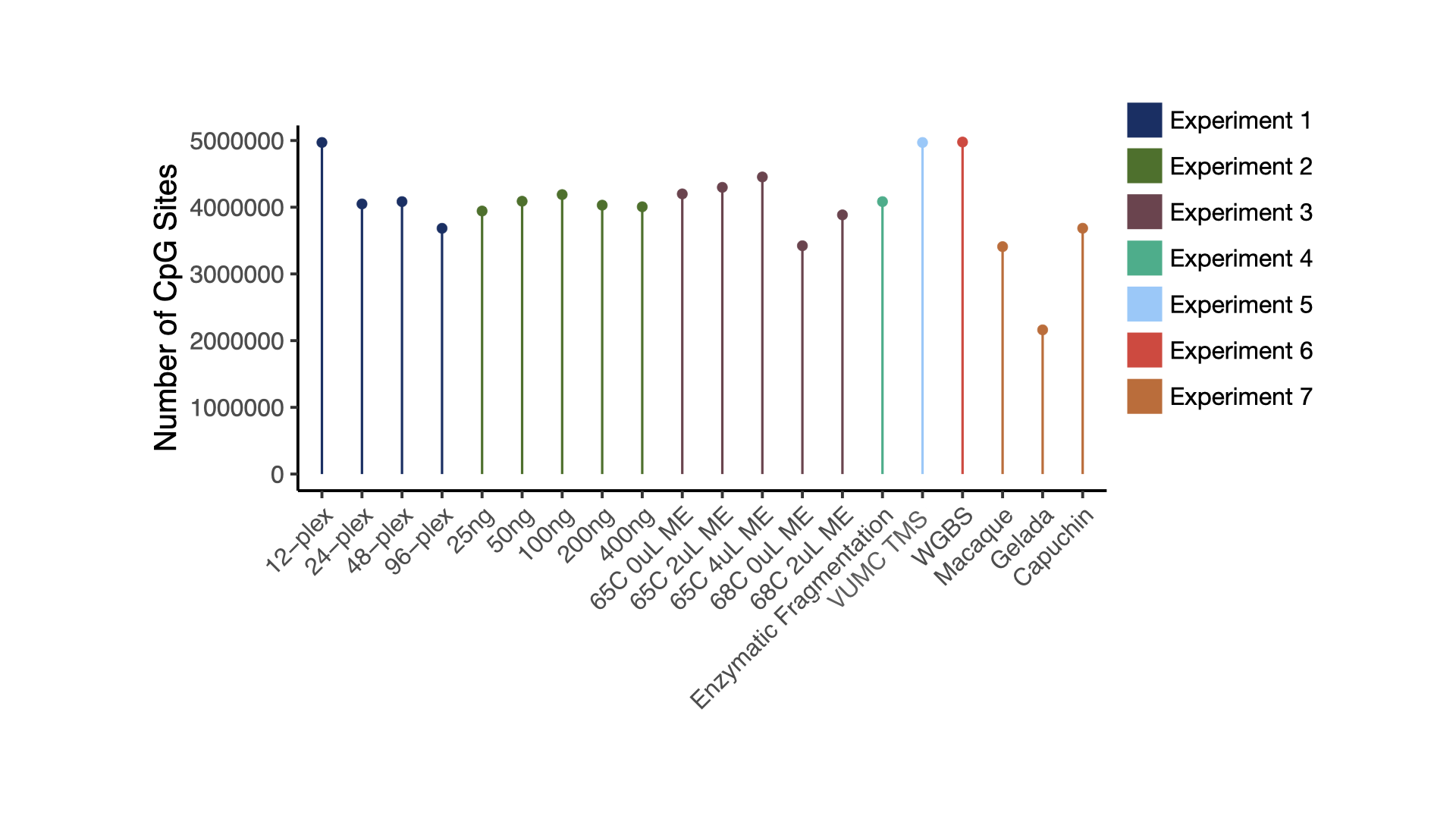

Supplement: S4 Fig — Number of CpG sites within 200 bp of target probes after filtering for>5X coverage in more than 75% of samples by experiment. Colors are representative of each experiment which are defined in Fig 1C. (TIFF) [file pgen.1011667.s021.tiff]

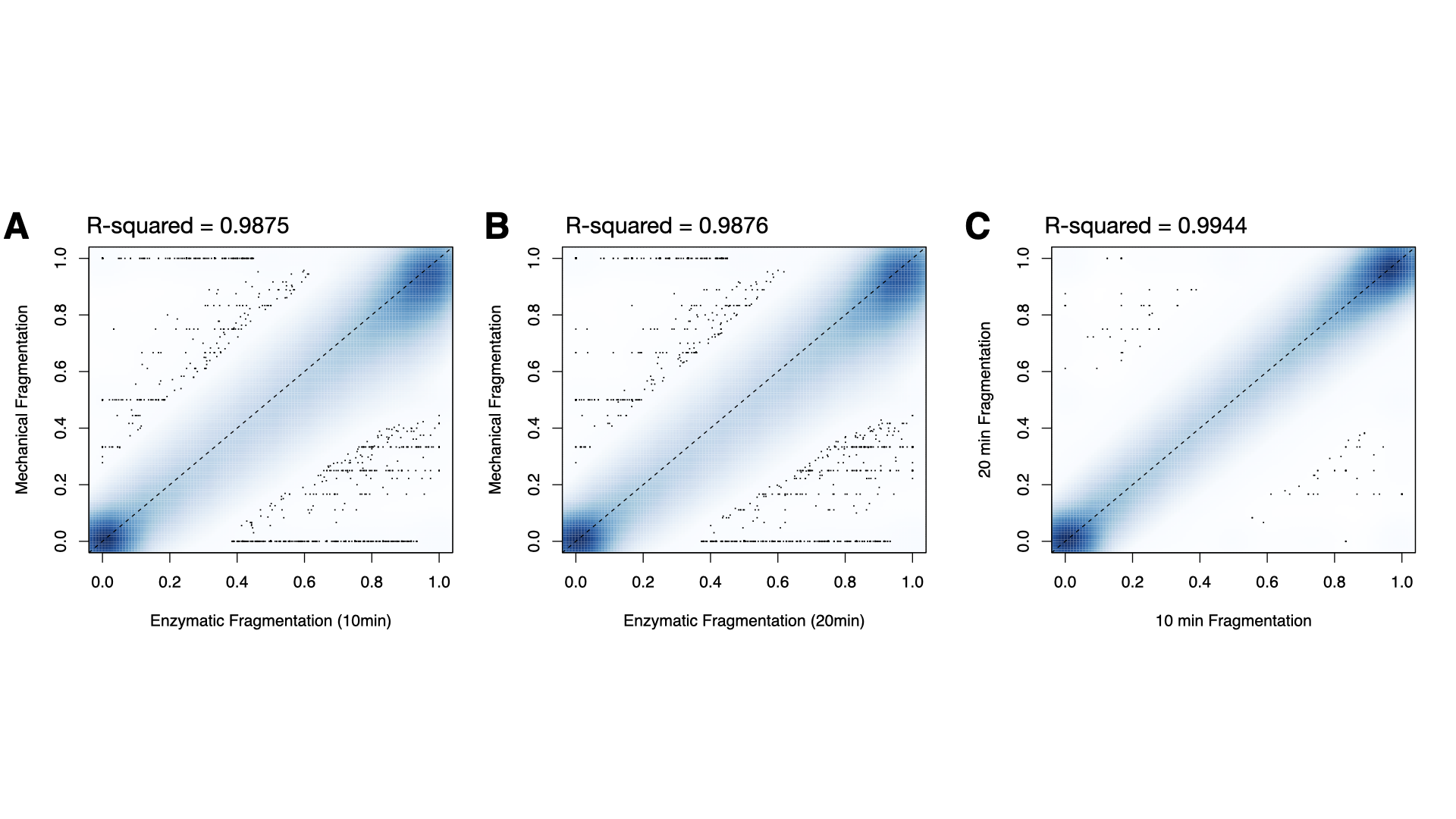

Supplement: S5 Fig — Site-level methylation averaged across 3 samples processed using mechanical fragmentation, enzymatic fragmentation for 10 minutes, and enzymatic fragmentation of 20 minutes. Each point represents a site measured across both fragmentation methods and R2 values were generated using linear modeling. (TIFF) [file pgen.1011667.s022.tiff]

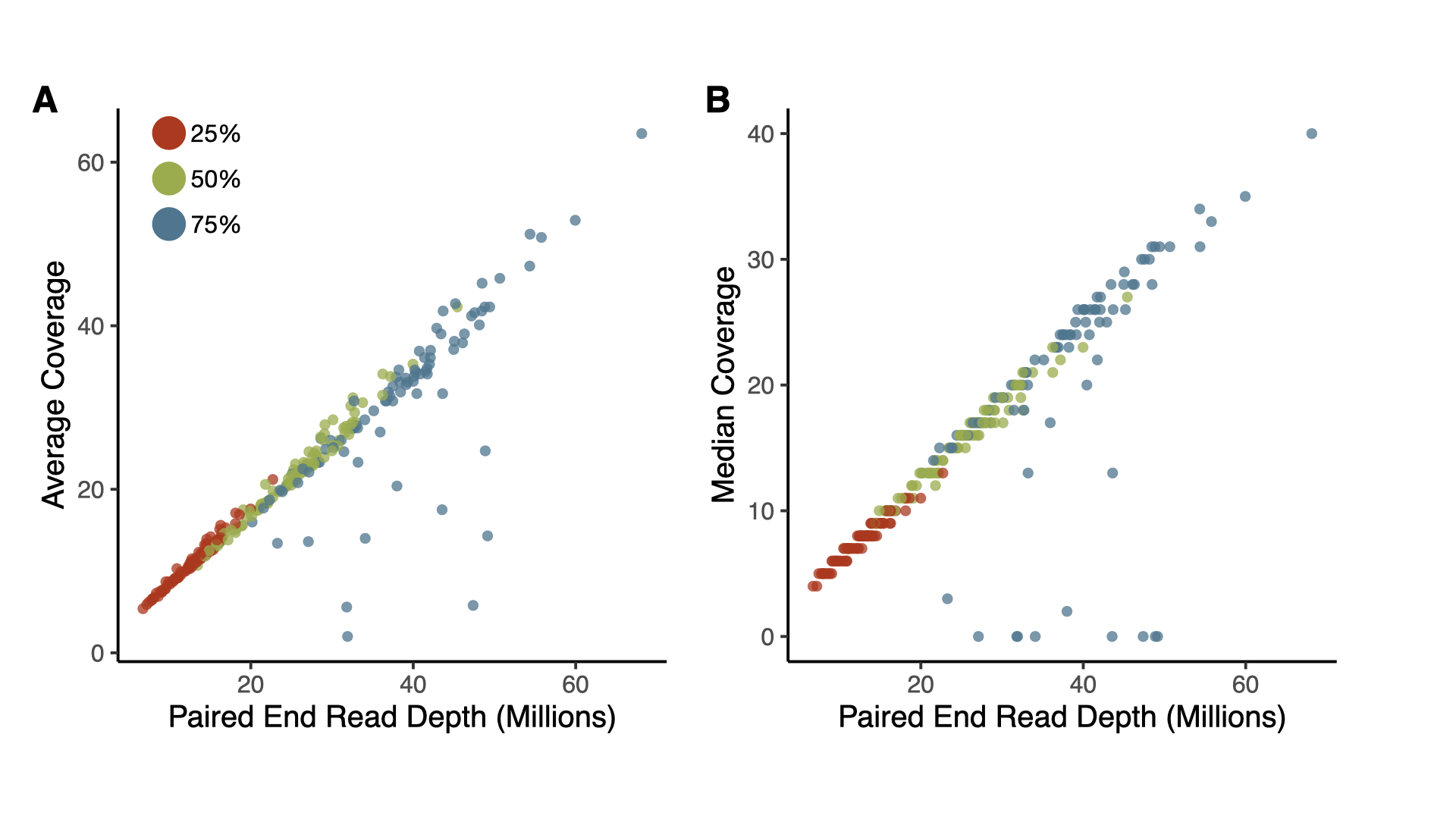

Supplement: S6 Fig — We subset the mapped read files for each sample (n = 88) included in our enzymatic fragmentation experiment (experiment 4) to include a random subset of 25, 50, or 75% of the total reads. We calculated the average (A) and median (B) coverage for on-target sites (y-axis) and observed a linear relationship between coverage and the number of subset reads (x -axis), which is useful for estimating what sequencing depth per sample will be needed to obtain various degrees of coverage. (TIFF) [file pgen.1011667.s023.tiff]

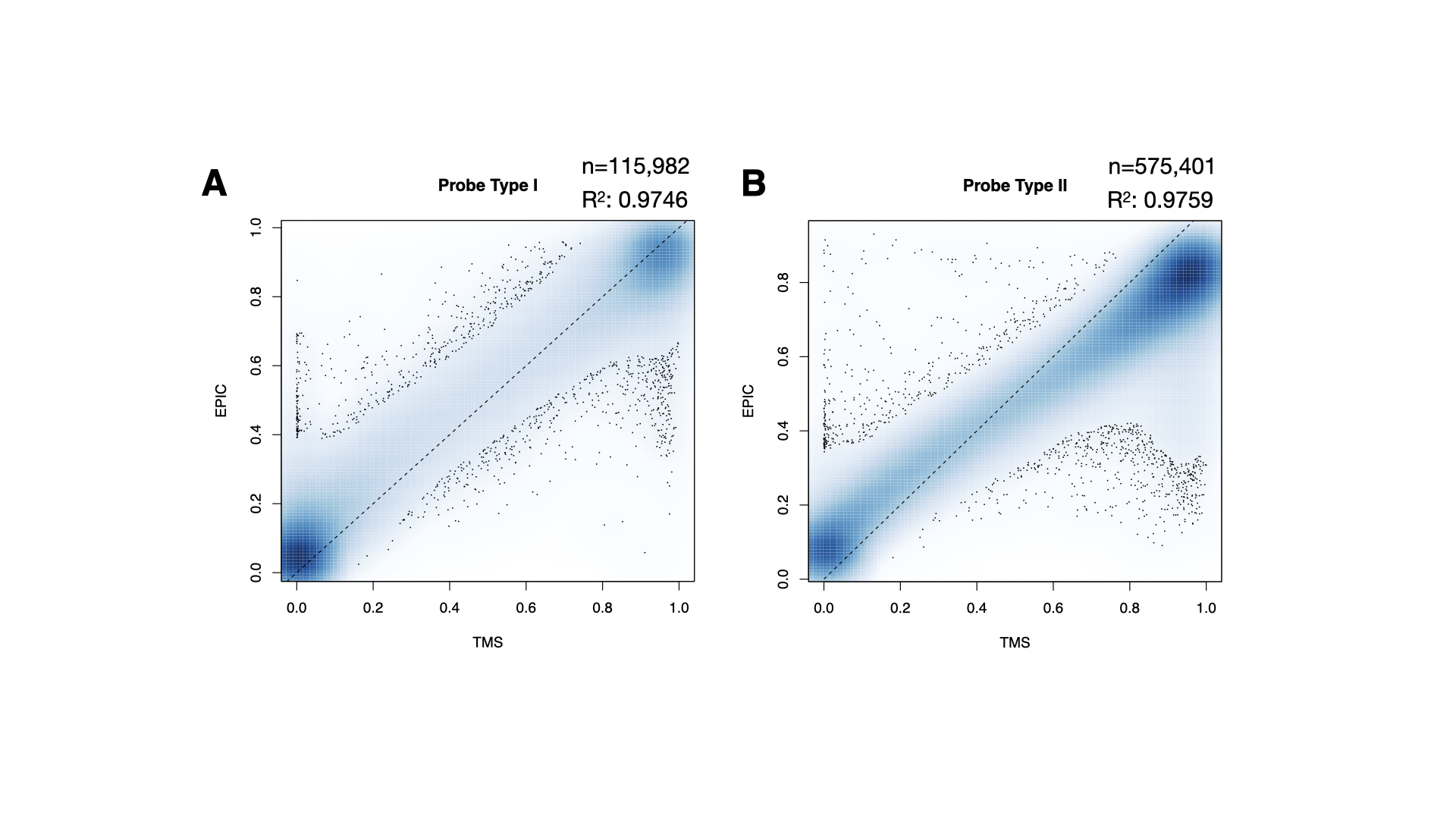

Supplement: S7 Fig — DNA methylation levels (A: n = 115,982 matched CpG sites; B: n = 575,401 matched CpG sites) averaged across 55 VUMC samples processed using TMS and the EPIC v2 array. Each point represents a site measured across both processing methods and R2 values were generated using linear modeling. (TIFF) [file pgen.1011667.s024.tiff]

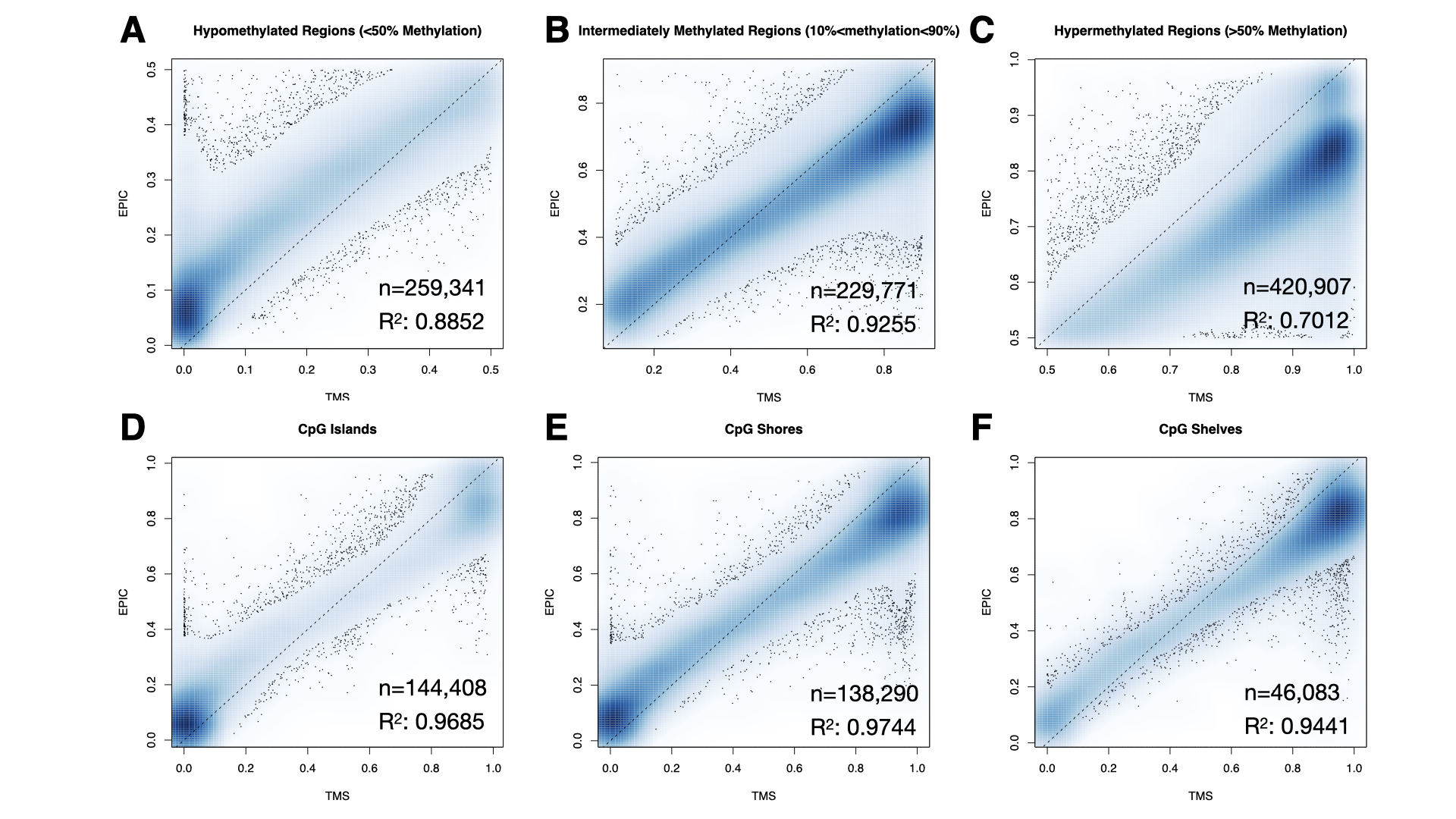

Supplement: S8 Fig — For samples processed using both TMS and the EPIC array (n = 55 VUMC samples), we assessed the correlation in site-level average methylation levels for (A) hypomethylated regions (<50% average methylation); (B) intermediately methylated regions (average methylation above 10% and below 90%); (C) hypermethylated regions (>50% methylation); (D) UCSC-annotated CpG islands; (E) UCSC-annotated CpG shores (i.e., regions within 2kb of the boundaries of a CpG island); and (F) UCSC-annotated CpG shelves (regions within 2kb and 4kb of the boundaries of a CpG island R2 values were calculated using linear modeling; sample sizes represent the number of CpG sites included in each panel. (TIFF) [file pgen.1011667.s025.tiff]

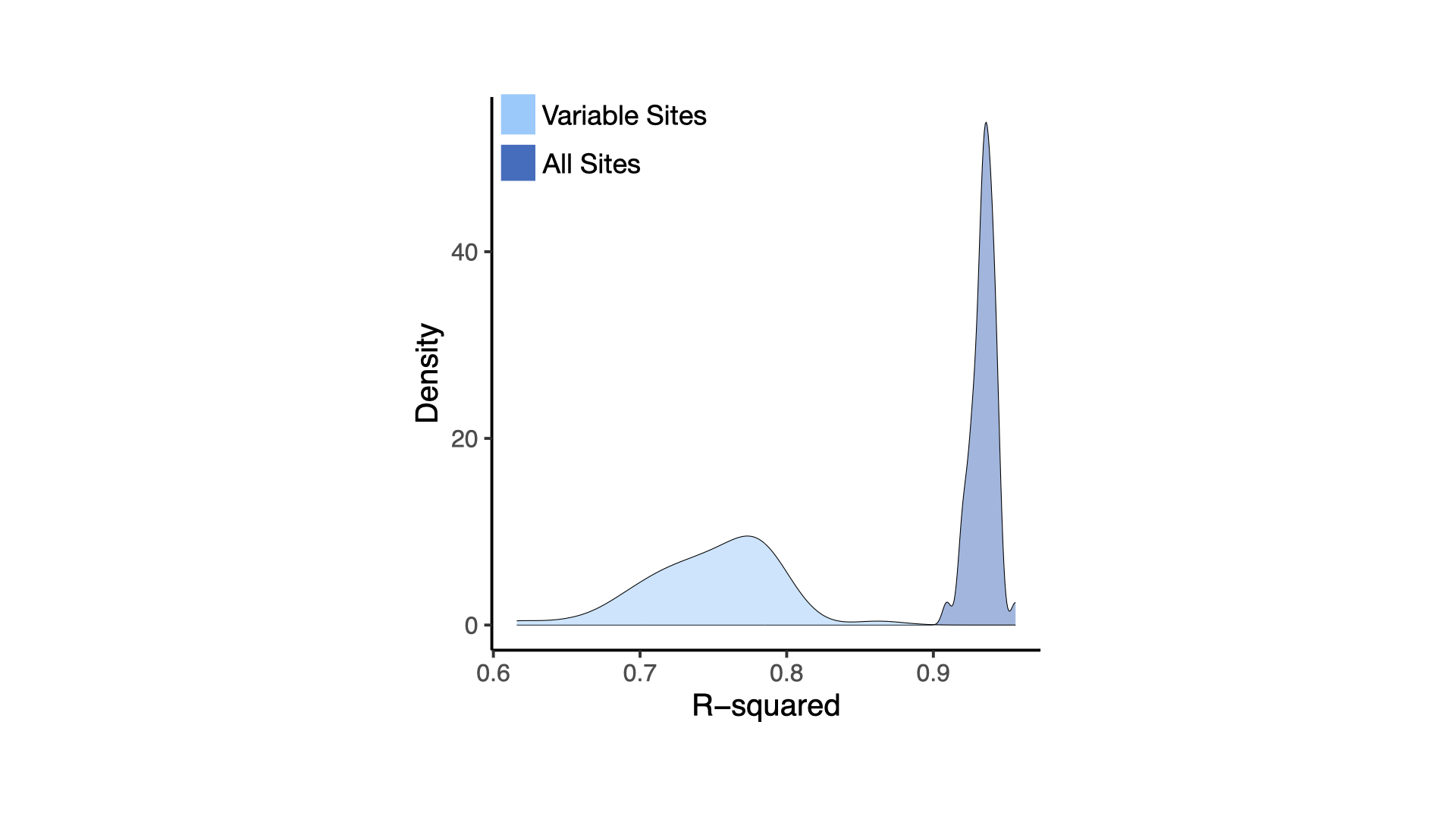

Supplement: S9 Fig — For samples processed using both TMS and the EPIC array, we assessed the correlation in site-level methylation for variable sites (methylation >0.1 and <0.9) and all sites after permuting sample ID randomly. R2 values were generated using linear modeling. (TIFF) [file pgen.1011667.s026.tiff]

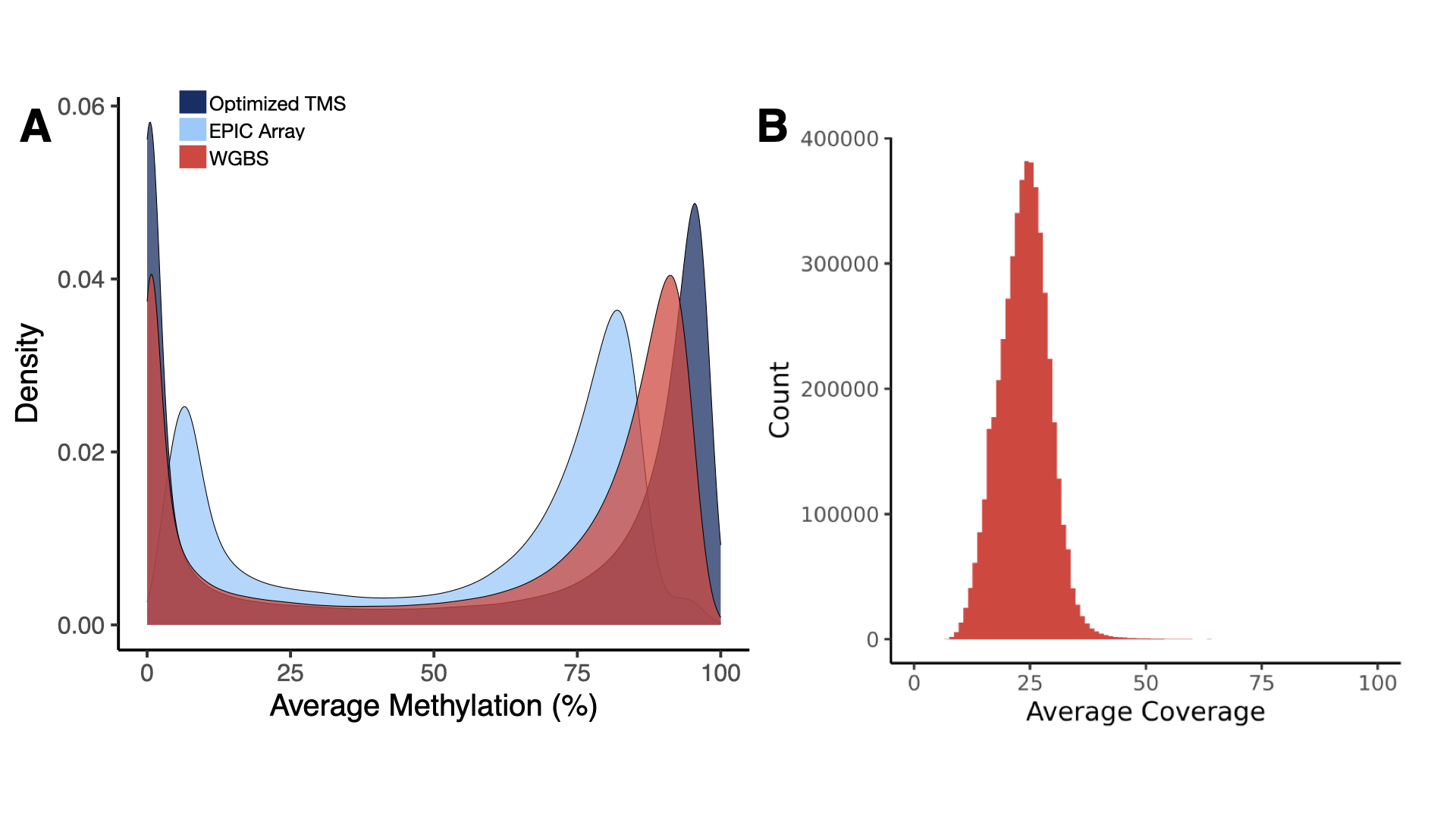

Supplement: S10 Fig — (A) Density plot showing the average methylation of a site (i.e., across samples) for filtered (>5X coverage in >75% of sites) sites captured between the three technologies (726,597 EPIC Array sites; 4,990,351 TMS sites; and 5,000,659 WGBS sites). Sites were not matched between the three technologies. (B) Average coverage per site of sites captured by WGBS after filtering for>5X coverage in >75% of samples. Median average coverage is 24.0X. (TIFF) [file pgen.1011667.s027.tiff]

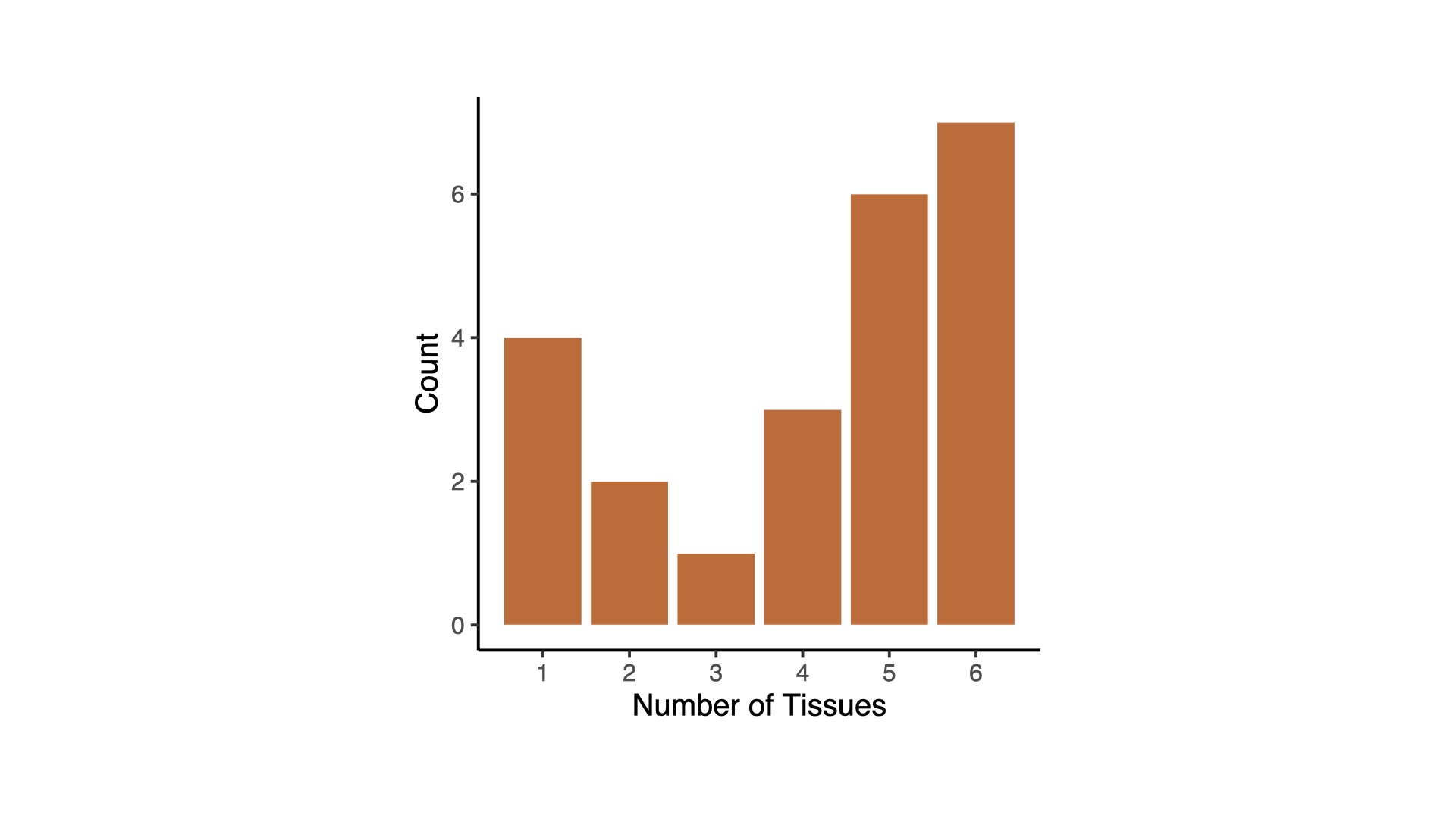

Supplement: S11 Fig — The majority of individuals had 4 + tissues represented in the dataset. (TIFF) [file pgen.1011667.s028.tiff]

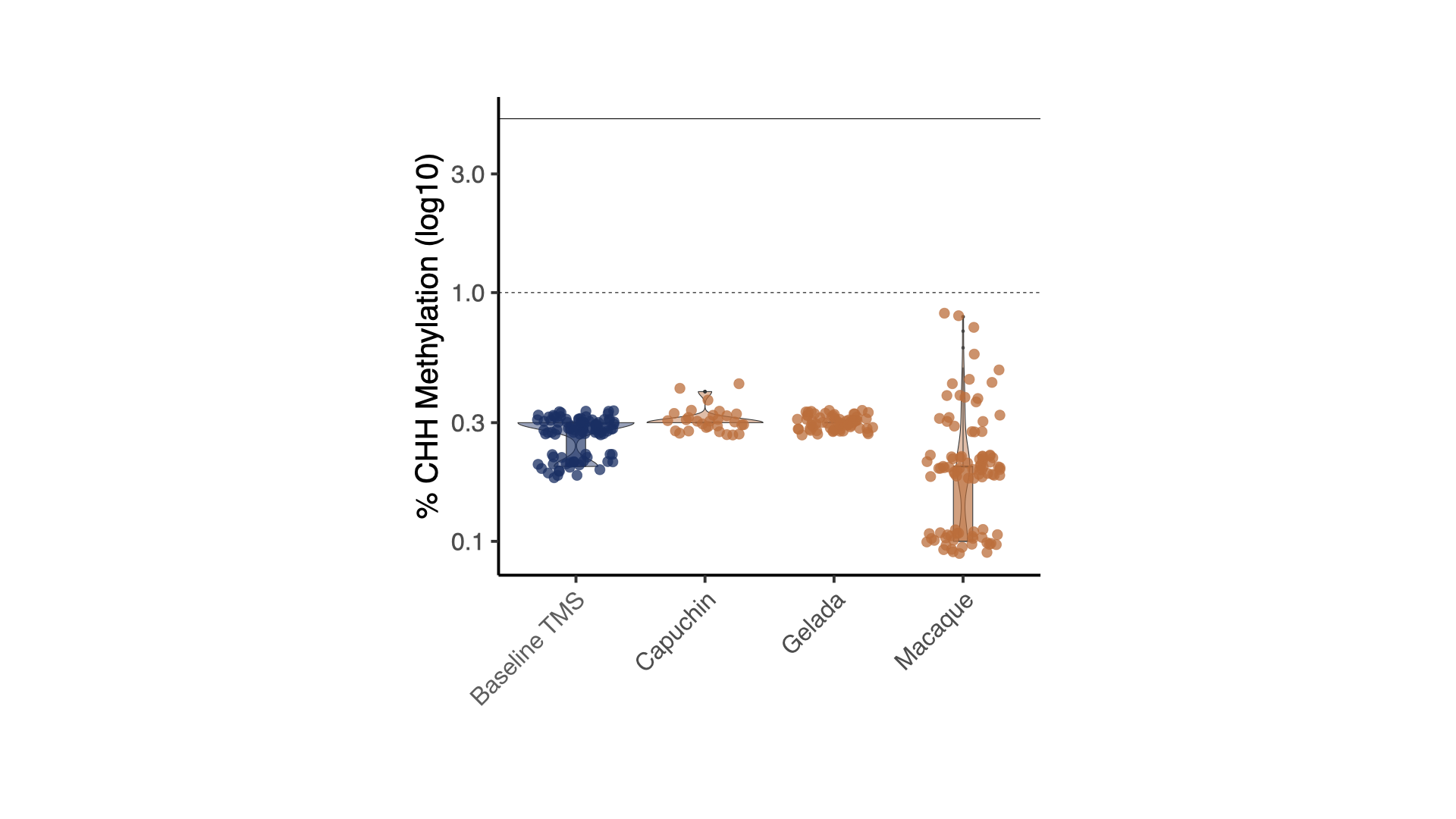

Supplement: S12 Fig — Percentage of cytosines in a CHH context marked as methylated (an estimate of conversion efficiency) following optimized TMS using genomic DNA from capuchins, geladas, and macaques. The dashed line refers to 1% CHH methylation and the solid line refers to 5% CHH methylation, a common cut off indicative of high levels of cytosine conversion. (TIFF) [file pgen.1011667.s029.tiff]

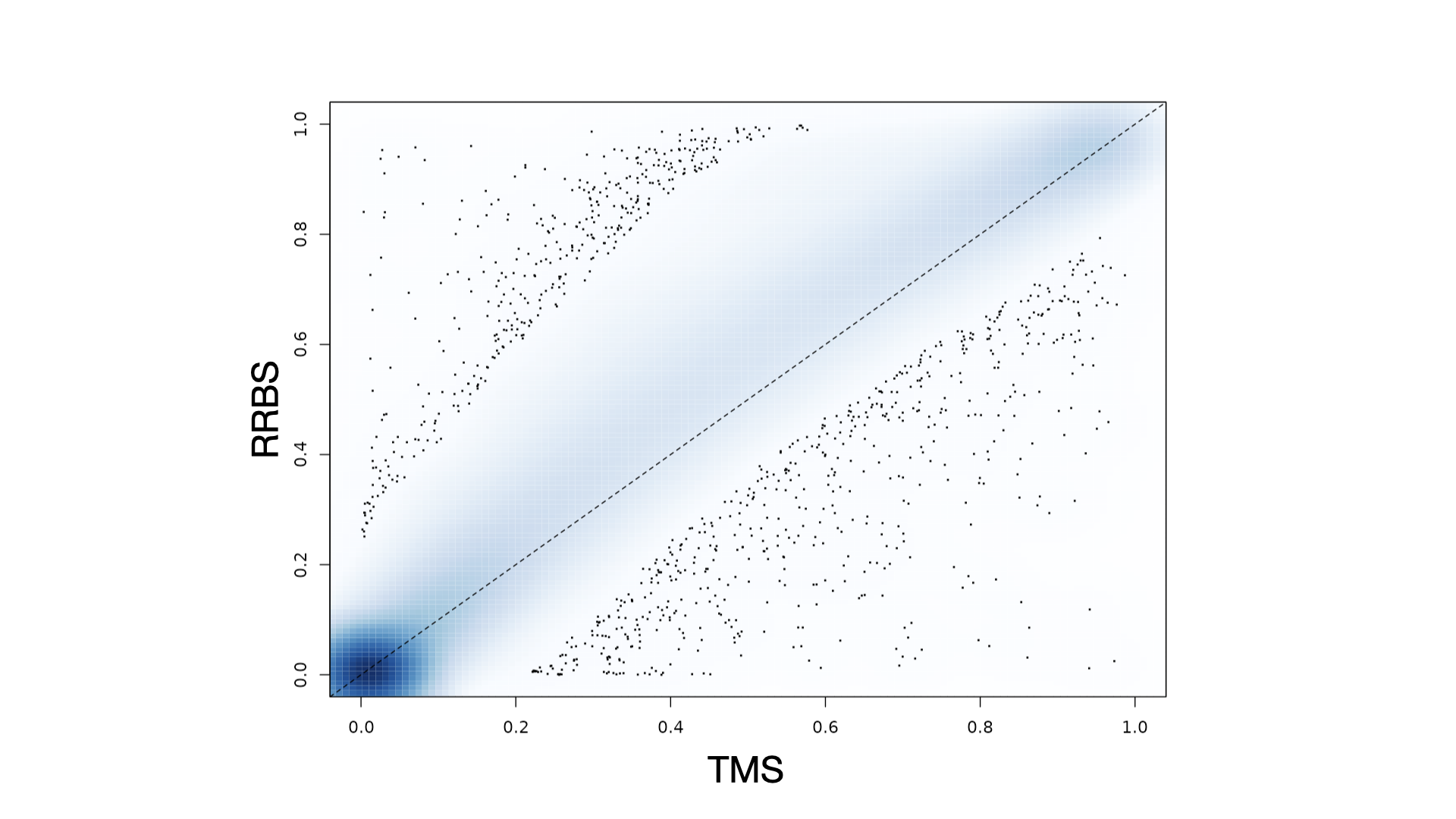

Supplement: S13 Fig — Site-level DNA methylation estimates averaged across 96 rhesus macaque samples processed using TMS and RRBS. Each point represents a site measured across both fragmentation methods and R2 values were generated using linear modeling. RRBS enriches for CpG dense regions of the genome, which tend to be hypomethylated. (TIF) [file pgen.1011667.s030.tif]

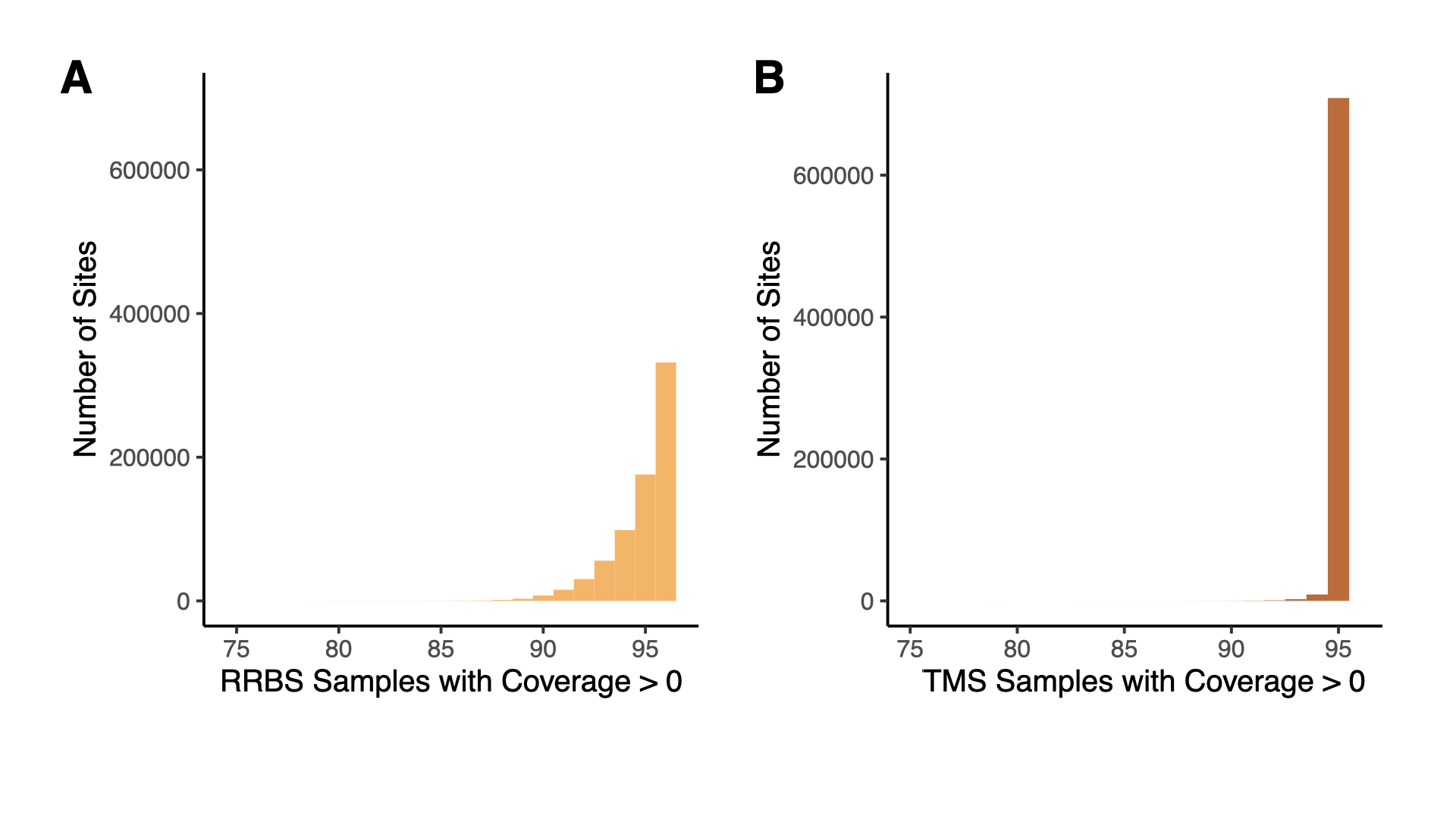

Supplement: S14 Fig — Sites filtered for>5X coverage in >75% of samples processed using a given technology. A greater number of sites are covered consistently across all 96 samples using TMS compared to RRBS. (TIFF) [file pgen.1011667.s031.tiff]

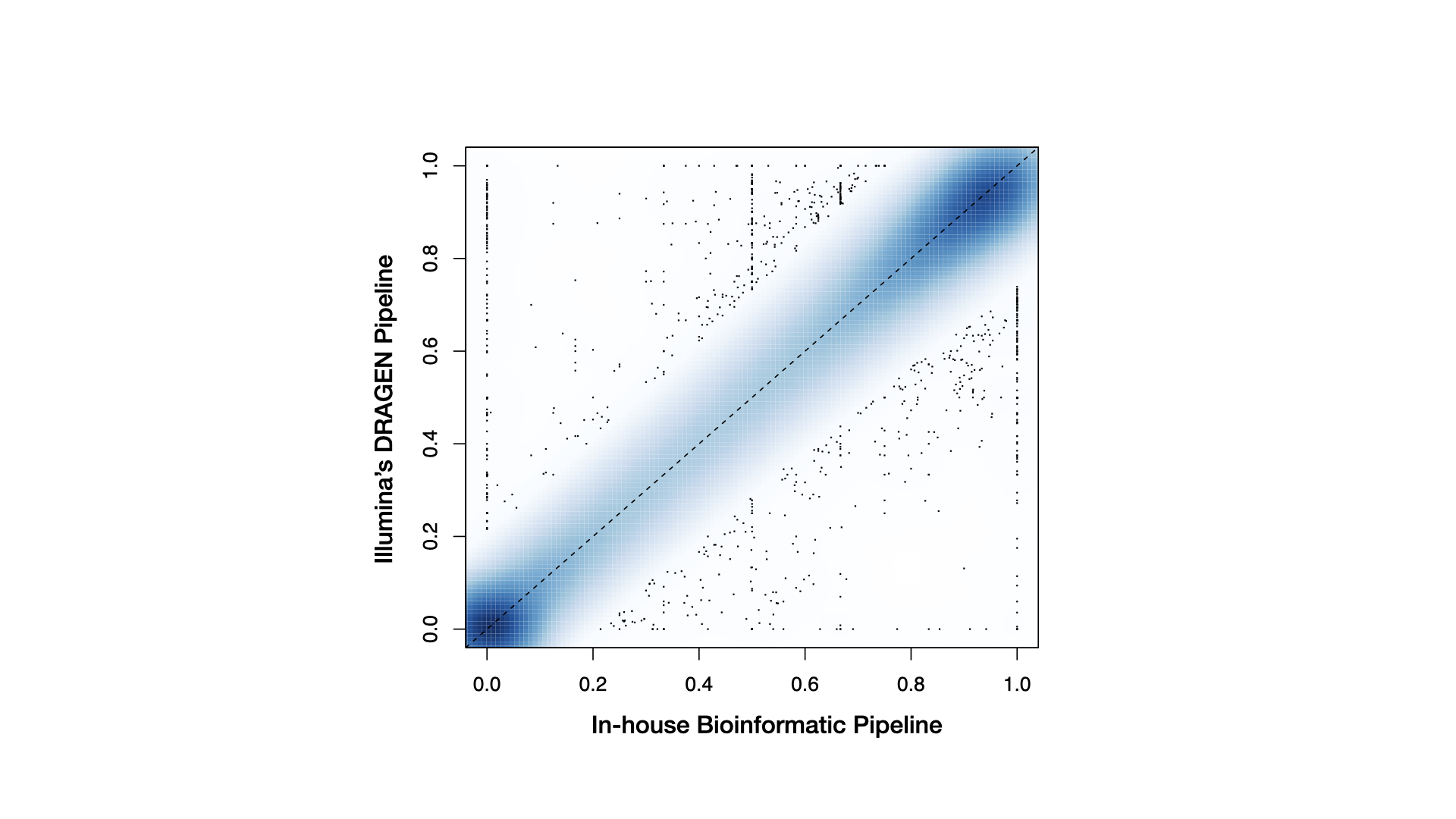

Supplement: S15 Fig — Each point represents the average methylation at a given site for 88 samples that were processed using both pipelines (R2 = 0.9972). (TIFF) [file pgen.1011667.s032.tiff]

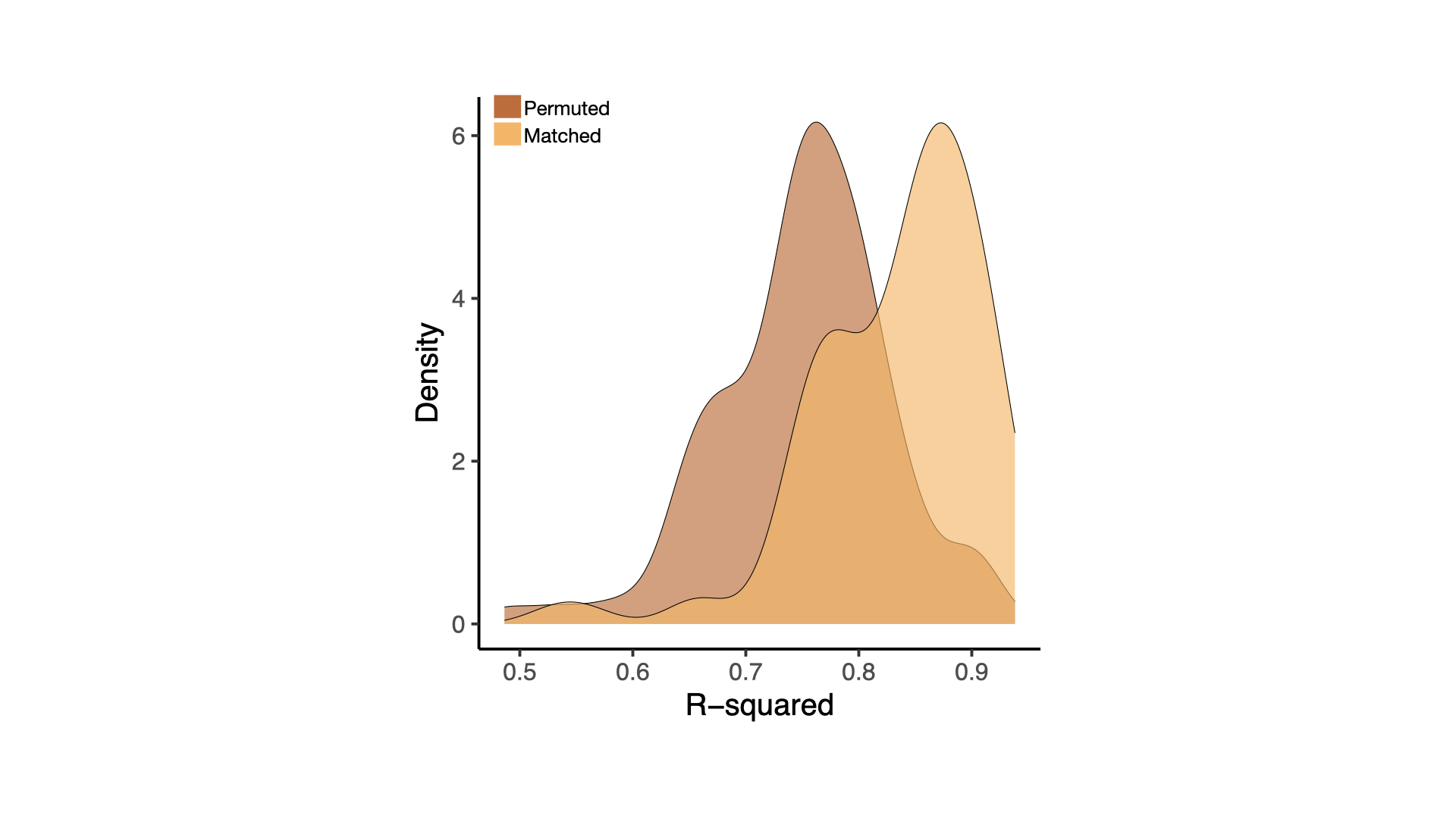

Supplement: S16 Fig — For samples processed using both TMS and RRBS, we assessed the correlation in site-level methylation for all sites after permuting sample ID randomly and compared them to non-permuted, or matched, sample IDs. R2 values were generated using linear modeling. Using a t.test, we found a significant difference between the means of the two samples (t = 7.6796, p-value = 8.224 x 10^–13^, mean of matched samples: 0.8345, mean of permuted samples: 0.7508). (TIFF) [file pgen.1011667.s033.tiff]
